# Deep behavioural phenotyping of the Q175 Huntington disease mouse model: effects of age, sex, and weight

**DOI:** 10.1186/s12915-024-01919-9

**Published:** 2024-05-23

**Authors:** Ellen T. Koch, Judy Cheng, Daniel Ramandi, Marja D. Sepers, Alex Hsu, Tony Fong, Timothy H. Murphy, Eric Yttri, Lynn A. Raymond

**Affiliations:** 1 Department of Psychiatry, Djavad Mowafaghian Centre for Brain Health, Vancouver, BC Canada; 2 Graduate Program in Neuroscience, University of British Columbia, Vancouver, BC Canada; 3 Graduate Program in Cell and Developmental Biology, University of British Columbia, Vancouver, BC Canada; 4 Department of Biological Sciences, Carnegie Mellon University, Pittsburgh, PA USA; 5 Present Address: Department of Clinical Neurosciences, Cumming School of Medicine, University of Calgary, Calgary, AB T2N 2T9 Canada

**Keywords:** Neuroscience, Behaviour, Huntington disease, Rotarod, Open field, T-maze, Motor skill, Motor learning, Striatum, Machine learning, Naturalistic behaviour; response learning

## Abstract

**Background:**

Huntington disease (HD) is a neurodegenerative disorder with complex motor and behavioural manifestations. The Q175 knock-in mouse model of HD has gained recent popularity as a genetically accurate model of the human disease. However, behavioural phenotypes are often subtle and progress slowly in this model. Here, we have implemented machine-learning algorithms to investigate behaviour in the Q175 model and compare differences between sexes and disease stages. We explore distinct behavioural patterns and motor functions in open field, rotarod, water T-maze, and home cage lever-pulling tasks.

**Results:**

In the open field, we observed habituation deficits in two versions of the Q175 model (zQ175dn and Q175FDN, on two different background strains), and using B-SOiD, an advanced machine learning approach, we found altered performance of rearing in male manifest zQ175dn mice. Notably, we found that weight had a considerable effect on performance of accelerating rotarod and water T-maze tasks and controlled for this by normalizing for weight. Manifest zQ175dn mice displayed a deficit in accelerating rotarod (after weight normalization), as well as changes to paw kinematics specific to males. Our water T-maze experiments revealed response learning deficits in manifest zQ175dn mice and reversal learning deficits in premanifest male zQ175dn mice; further analysis using PyMouseTracks software allowed us to characterize new behavioural features in this task, including time at decision point and number of accelerations. In a home cage-based lever-pulling assessment, we found significant learning deficits in male manifest zQ175dn mice. A subset of mice also underwent electrophysiology slice experiments, revealing a reduced spontaneous excitatory event frequency in male manifest zQ175dn mice.

**Conclusions:**

Our study uncovered several behavioural changes in Q175 mice that differed by sex, age, and strain. Our results highlight the impact of weight and experimental protocol on behavioural results, and the utility of machine learning tools to examine behaviour in more detailed ways than was previously possible. Specifically, this work provides the field with an updated overview of behavioural impairments in this model of HD, as well as novel techniques for dissecting behaviour in the open field, accelerating rotarod, and T-maze tasks.

**Supplementary Information:**

The online version contains supplementary material available at 10.1186/s12915-024-01919-9.

## Background

Huntington disease (HD) is a progressive neurodegenerative disorder characterized by motor, cognitive, and psychiatric disturbances [1, 2]. It is caused by an autosomal dominant CAG repeat expansion in the Huntingtin (Htt) gene, resulting in aberrant forms of the Htt protein [1]. Many direct and indirect effects of the mutant Htt (mHtt) protein have been identified in humans and animal models, including changes to gene expression, synaptic transmission, and intracellular signaling [1]. On the macroscopic scale, HD causes extensive degeneration of the dorsal striatum, in addition to progressive degeneration of other brain regions including cortex [2].

Due to the monogenic nature of HD, many animal models have been created, contributing to discoveries in HD research. Among these, genetic mouse models are the most developed and commonly used (reviewed in [3]). These mouse models vary in the severity of phenotype, including the alterations in brain structural and electrophysiological measures, as well as behavioural changes they exhibit. Although none of the mouse models recapitulate all the brain and behavioural changes seen in human HD, especially the cortical atrophy and profound striatal volume loss, most of these models show progressive motor and cognitive deficits, such as impaired rotarod learning, changes to spontaneous behaviour patterns, and impaired learning in T-maze tasks [4–6]. These impairments mimic changes that occur in humans, including altered motor learning and coordination, and cognitive deficits, although the timing and degree of impairment in these mice varies widely between models.

Knock-in mouse models have CAG repeat expansions of various lengths expressed in the mouse Htt locus [3, 6–8], and have gained popularity due to their accuracy in replicating the genetic context that occurs in HD patients [3]. The zQ175 model contains a knock-in of 175 CAG repeats into the mouse Htt allele on a C57BL/6 J background [6, 9] and was derived from a spontaneous CAG expansion in the CAG140 knock-in colony [8]. To further improve the phenotype of this model, newer versions of zQ175 mice have been developed in which a floxed neomycin selection cassette is excised (zQ175dn), resulting in increased expression of the mHtt transgene [10, 11]. Studies have characterized physiological and behavioural changes in zQ175dn mice that progress with age; however, these are often subtle, vary by sex and background strain, and are more apparent in homozygous than heterozygous mice [6, 9, 10]. Given that the vast majority of HD-affected individuals carry a single copy of the mutant gene [12], it is crucial to use heterozygous mice when employing knock-in models to accurately reflect the genetic environment in which HD develops.

In this study, we investigate the behaviour of heterozygous male and female zQ175dn mice at two ages: 2–3-month-old (“premanifest”) mice and 9–12-month-old (“manifest”) mice. Recent work from our group has revealed interesting behavioural deficits in the YAC128 mouse model, such as altered paw kinematics on the accelerating rotarod, even in mice that were performing normally on the task according to traditional measures [13]. In this study, we have combined machine learning-based analysis approaches [14, 15] to characterize behaviour on the rotarod, open field, water T-maze, and a home cage-based lever pulling assay in the zQ175dn mouse model. We compare and contrast behaviour at premanifest and manifest disease stages, between males and females, as well as to open field behaviour of the Q175FDN model, which differs in background strain. We also present electrophysiological measures for select experiments. Our work highlights new ways that behaviour can be characterized in mice that is particularly useful in identifying subtle behavioural changes and provides a comprehensive report of how these behaviours are affected in the zQ175dn model.

## Results

### Manifest Q175 mice show impaired habituation and altered behavioural patterns in the open field

**Fig. 1:**
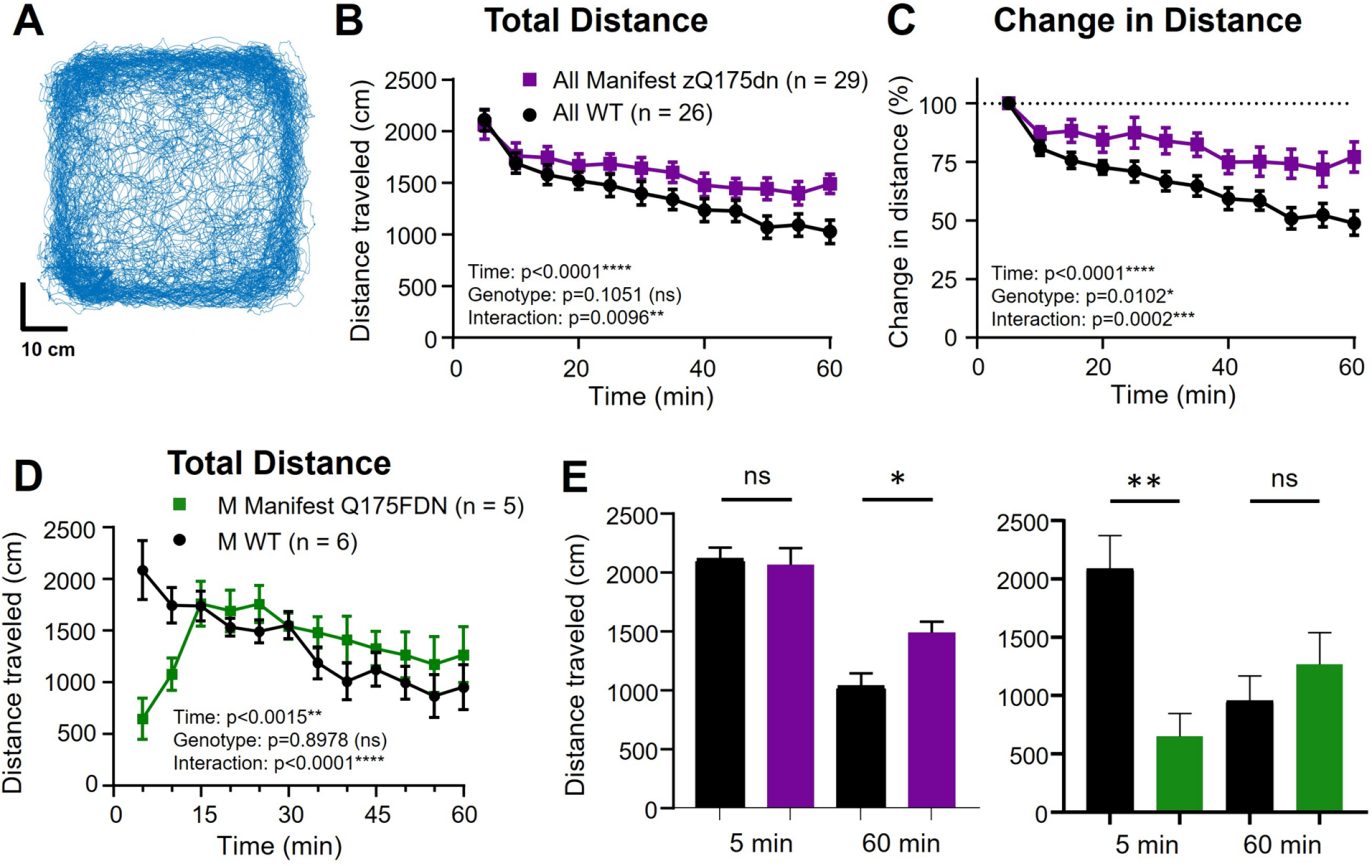
Total distance traveled and percentage change in distance traveled per 5-min intervals during a single 60-min open field trial in manifest zQ175dn and Q175FDN compared to wild-type (WT) littermates. A) Example of mouse tracking of the base of the tail in the open field for 60 min. B) Total distance traveled per 5-min interval in the open field for manifest zQ175dn mice (males and females combined). C) Percentage change in distance traveled compared to the first 5-min interval in the open field for manifest zQ175dn mice (males and females combined). D) Total distance traveled per 5-min interval in the open field for male manifest Q175FDN mice. E) Distance traveled in the first 5 min (5 min) and last 5 min (60 min) for male manifest zQ175dn mice and WT littermates (left panel; genotype effect, last 5 min, *p* = 0.0141*) and Q175DFN mice and WT littermates (right panel; genotype effect, first 5 min, *p* = 0.0014**). One-way or two-way analysis of variance was used for all statistical analysis Asterisks (*) denote significance level. Individual values for groups with *n* < 6 are provided in Additional file 7: Individual values. ns = not significant. M = Male

All groups of zQ175dn and Q175FDN mice and their respective WT controls completed a single one-hour open field trial (Fig. 1A) to assess naturalistic behaviour as mice explore a novel space. Manifest male and female zQ175dn mice showed a deficit in open field habituation, as indicated by a smaller reduction in distance traveled over time (Fig. 1B, C, E; Additional file 1: Fig S1A, B), while male manifest Q175FDN mice exhibited a unique pattern of behaviour in the open field, with significantly lower distance traveled in the first 10 min of the trial which then rose to WT levels for the final 50 min (Fig. 1D, E). Premanifest male and female zQ175dn mice exhibited similar distance traveled to WT littermates (Additional file 1: Figure S1C). Other measures such as percentage of time at center did not show a difference between genotypes for any groups (manifest zQ175dn: 17.0 ± 1.6 percent, WT littermates: 15.6 ± 1.6 percent, *p* = 0.5225; premanifest zQ175dn: 19.8 ± 2.2 percent, WT littermates: 18.1 ± 1.8 percent, *p* = 0.5415 2-way ANOVA; males and females pooled).

**Fig. 2:**
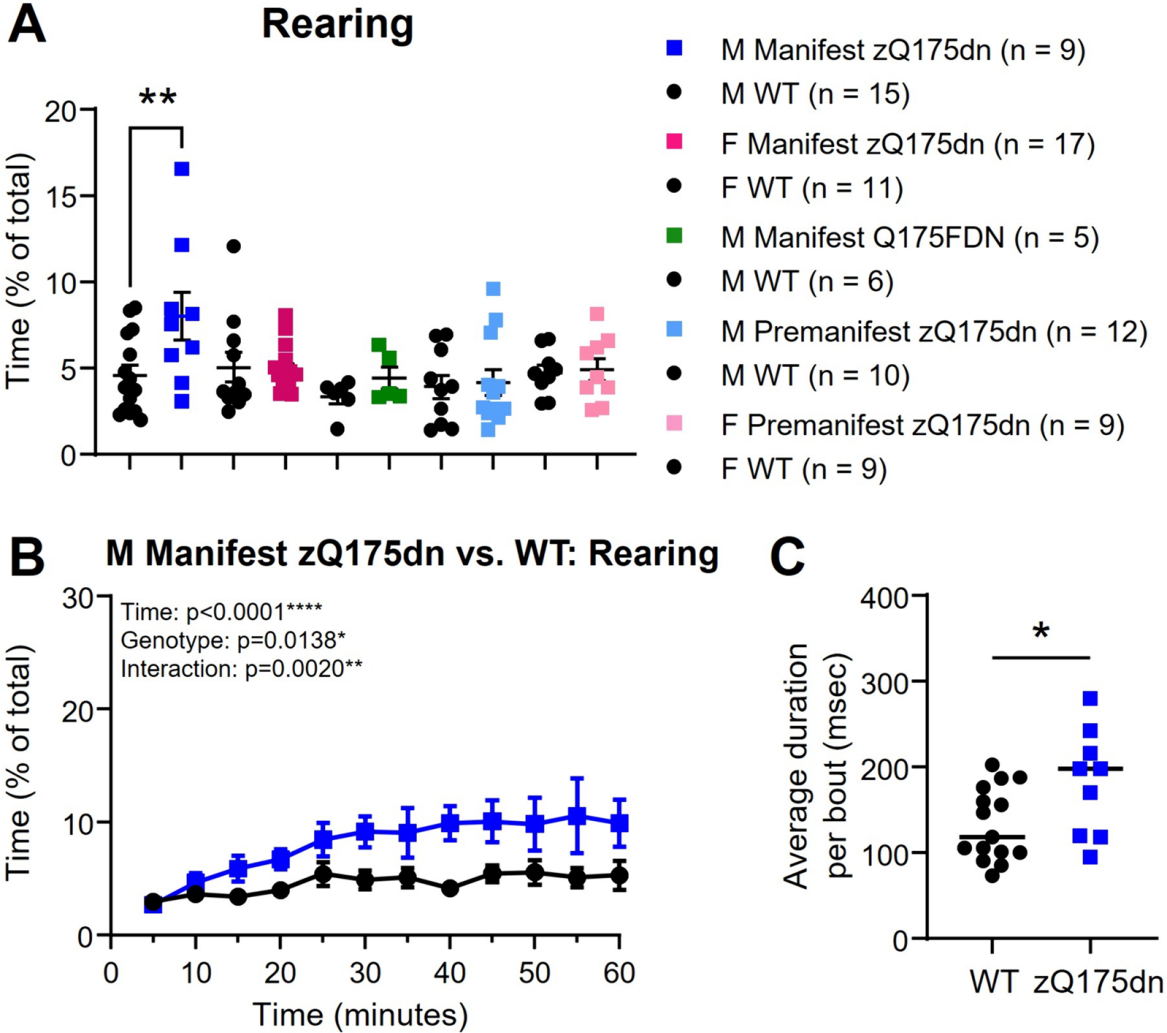
Engagement in rearing behaviour (away from the wall) during a single 60-min open field trial in Q175 mice compared to WT littermates. **A** Percentage of time spent rearing away from the wall (overall effect, *p* = 0.0117*; genotype effect for male manifest zQ175dn vs. WT littermates, *p* = 0.0031**; one-way ANOVA with multiple comparisons). **B** Percentage of time spent rearing in 5-min intervals over the 60-min trial in male manifest zQ175dn mice (mixed effects analysis). **C** Average duration of each bout of rearing per mouse in male manifest zQ175dn mice (genotype effect, *p* = 0.0154*; unpaired t-test). Asterisks (*) denote significance level. Individual values for groups with *n* < 6 are provided in Additional file 7: Individual values. ns = not significant. M = Male. F = Female

We used the unsupervised machine learning algorithm B-SOiD [14] to further investigate how behaviour is affected in the open field. Using this method, we were able to reliably detect occurrences of locomotion (walking forward), turning left, turning right, sniffing, and rearing behaviours (Fig. 2, Additional file 2: Figure S2). Locomotion and turning behaviours were combined in our analysis as mice generally alternated between these behaviours. All groups spent the largest proportion of time in locomotion and turning behaviours (range of means for groups: 30 – 37% of the hour) and sniffing behaviours (range of means: 18 – 32% of the hour) with no genotype differences (Additional file 2: Figure S2A, B [top panels]). Rearing (away from the wall) accounted for a smaller proportion of total time (range of means: 3 – 8% of the hour), with male manifest zQ175dn mice showing increased rearing compared to WT littermates (Fig. 2A). Over time in the open field session, male manifest zQ175dn increased their total percentage of time rearing (Fig. 2B) and showed a longer average duration for each bout of rearing (Fig. 2C) compared to WT littermates.

Although rearing in male manifest zQ175dn was the only behaviour to show a significant genotype difference when we analyzed total engagement in this behaviour, other differences appeared when we explored engagement in behaviours over time. Over the hour in the open field, WT littermates for all groups showed a reduction in locomotion and turning behaviours (Additional file 2: Figure S2A). Female manifest zQ175dn also showed a reduction, though smaller, in this behaviour over time, with a significant interaction between time and genotype for locomotion and turning, while males did not (Additional file 2: Figure S2A). Notably, male manifest Q175FDN mice engaged in locomotion and turning less during the first 10 min in the open field, and engaged in sniffing behaviours more, compared to WT littermates (Additional file 2: Figure S2A, B), which may indicate increased anxiety in these mice. Male manifest zQ175dn mice showed a decrease in sniffing over the hour while WT littermates showed a slight increase in sniffing; female manifest and both pre-manifest zQ175dn groups showed no difference in sniffing behaviour (Additional file 2: Figure S2B).

Altogether, our open field behavioural analysis revealed differences in behaviour patterns of Q175 mice that differ by sex and background strain.

### Rotarod performance is affected by protocol and animal weight

The rotarod was used to assess motor learning and kinematics in zQ175dn mice. We tested two protocols for the accelerating rotarod task, which we called “Rotations Allowed” and “Standard” (described in methods) in the male manifest zQ175dn mice and their WT littermates. We found no difference in time on rotarod or time to first rotation between zQ175dn mice and WT on the Rotations Allowed protocol (Additional file 3: Figure S3A). Surprisingly, we also found no change in time on rotarod or time to first rotation over days of training for either genotype (Additional file 3: Figure S3A), indicating that animals were not improving with training on this version of the task. Consequently, we did not continue with the Rotations Allowed protocol for other groups. On the Standard protocol, all groups tested improved in time on rotarod over days of training, and interestingly, male manifest zQ175dn mice performed significantly better than their WT littermates, while female manifest zQ175dn mice performed equally well to littermates (Additional file 3: Figure S3C, D).

**Fig. 3:**
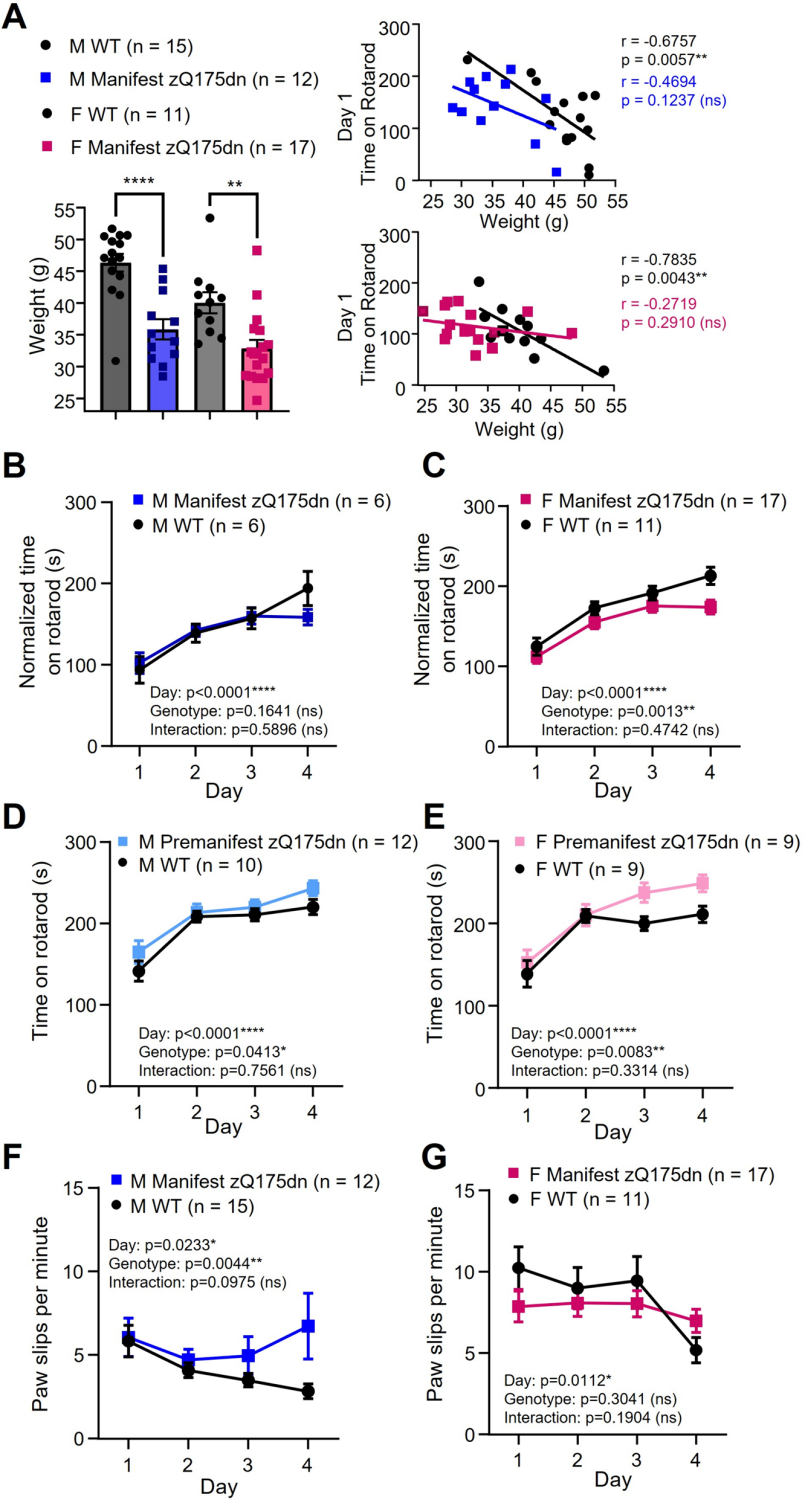
Rotarod performance and effects of weight in zQ175dn mice and wild-type (WT) littermates. **A** Left panel: Weight (g) on day 1 of rotarod training in male and female manifest zQ175dn mice compared to WT littermates (overall genotype effect, *p* < 0.0001****; genotype effect for male manifest zQ175dn vs. WT littermates (multiple comparisons), *p* < 0.0001****; genotype effect for female manifest zQ175dn vs. WT littermates, *p* = 0.0043**; two-way analysis of variance [ANOVA] with multiple comparisons). Right panels: Correlation between weight (g) and average time on rotarod on day 1 of training for manifest zQ175dn mice and WT littermates (Pearson’s *r* correlation test). **B** Time on rotarod normalized by weight (see methods for details) for male manifest zQ175dn mice and WT littermates that performed the Standard Protocol. **C** Time on rotarod normalized by weight for female manifest Q176/B6 mice and WT littermates. **D** Time on rotarod for male premanifest zQ175dn mice and WT littermates. **E** Time on rotarod for female premanifest zQ175dn mice and WT littermates. **F** Average number of paw slips per minute for male manifest zQ175dn mice and WT littermates. **G** Average number of paw slips per minute for female manifest zQ175dn mice and WT littermates. Two-way ANOVA used for all statistical analysis unless otherwise noted. Asterisks (*) denote significance level. ns = not significant. M = Male. F = Female

In light of these puzzling results, we turned our attention to animal weight and its effects on rotarod performance. Our male manifest zQ175dn mice were on average 10.48 ± 2.09 g (g) lighter than WT littermates (genotype: *p* < 0.0001****; zQ175dn range: 28.5—45.4 g; WT range: 30.9—51.7 g), and female manifest zQ175dn mice were 7.205 ± 2.17 g lighter than WT littermates (genotype: *p* = 0.0027**; zQ175dn range: 24.7—48.3 g; WT range: 33.6—45.4 g; Fig. 3A). Both male and female WT mice showed a significant inverse correlation between time on rotarod and weight on day 1 of training, while manifest zQ175dn mice of either sex did not show this correlation (Fig. 3A). After normalizing for weight (see methods), we found that male manifest zQ175dn mice showed a modest yet significant reduction in time on rotarod compared to WT littermates on the Rotations Allowed protocol (Additional file 3: Figure S3B). On the Standard protocol, male manifest zQ175dn performed equally well to WT for time on rotarod after weight normalization (Fig. 3B) in contrast to performing better than WT before weight normalization (Additional file 3: Figure S3C). Female manifest zQ175dn performed significantly worse than WT littermates for normalized time on rotarod on the Standard protocol (Fig. 3C). As expected, male and female premanifest zQ175dn mice did not show any deficit on rotarod and actually performed better than WT littermates (Standard protocol; Fig. 3D, E), and there was no difference in weight between genotypes at this age (*p* = 0.6456; unpaired t-test; males and females pooled).

### Male, but not female, manifest zQ175dn mice show altered paw kinematics on the rotarod

Previously, our group developed a method to examine paw kinematics on the rotarod, by tracking the vertical position of the paws relative to the rotarod [13]. Here, we found increased paw slip (when one or both paws dip below the bottom of the rotarod, not normalized by weight) frequency in the male manifest zQ175dn mice, particularly on day 4 of training (Fig. 3F). In contrast, female manifest zQ175dn mice did not show any difference in paw slip frequency (Fig. 3G), and both male and female premanifest mice showed similar paw slip frequencies to WT littermates (male premanifest zQ175dn: 8.08 ± 0.50 slips/minute, WT littermates: 7.54 ± 0.26 slips/minute, genotype effect, *p* = 0.5228; female premanifest zQ175dn: 2.95 ± 0.73 slips/minute, WT littermates: 2.90 ± 0.49 slips/minute, *p* = 0.9479; 2-way ANOVA).

### Water T-maze performance is impaired in zQ175dn mice and affected by animal weight

**Fig. 4:**
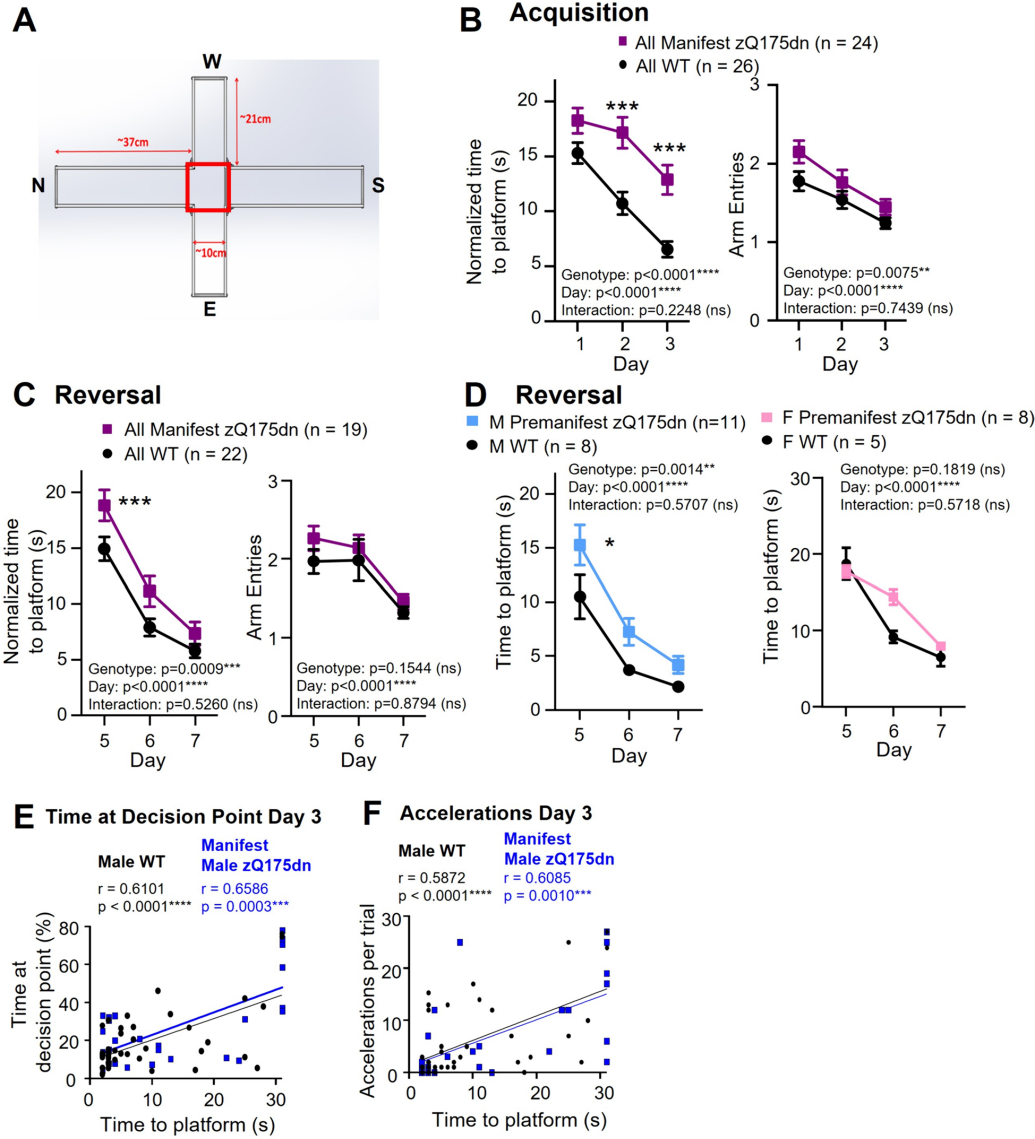
Water T-maze performance and behavioural feature analysis in zQ175dn mice and WT littermates. **A** Schematic of the water T-maze with red box outlining the area defined as the “decision point” for the behavioural feature analysis. **B** Left panel: Time to platform normalized by weight (see methods for details) for manifest zQ175dn mice and WT littermates (males and females) during the acquisition phase. Right panel: Number of arm entries for manifest zQ175dn mice and WT littermates (males and females) during the acquisition phase. **C** Left panel: Time to platform normalized by weight for manifest zQ175dn mice and WT littermates during the reversal phase. Right panel: Number of arm entries for manifest zQ175dn mice and WT littermates during the reversal phase. **D** Time to platform during the reversal phase for male (left panel) and female (right panel) premanifest zQ175dn mice compared to WT littermates. **E** Correlation between percentage of time at decision point and time to platform (not normalized) for day 3 of acquisition (Pearson’s *r* correlation test) in male manifest zQ175dn mice and WT littermates. **F** Correlation between number of accelerations per trial and time to platform (not normalized) for day 3 of acquisition (Pearson’s *r* correlation test) in male manifest zQ175dn mice and WT littermates. Note: due to a flash drive error, 2 female manifest zQ175dn and 5 WT littermates were excluded from analysis for days 1 and 2 of acquisition for water T-maze experiments (therefore for these two days *n* = 22 for female manifest zQ175dn and *n* = 21 for WT littermates). See methods for details. Asterisks (*) denote significance level. Individual values for groups with *n* < 6 are provided in Additional file 7: Individual values. ns = not significant. M = Male. F = Female

zQ175dn animals performed a water T-maze task (Fig. 4A) in which they located a hidden platform in one of two arms, to assess response learning and reversal learning. Interestingly, animal weight and time to platform on day 1 of the acquisition phase was positively correlated in the 9 – 11-month old male and female WT mice (males: *r* = 0.6239, *p* = 0.0129*; females: *r* = 0.8853, *p* = 0.0190*; Pearson’s *r* correlation), whereas this correlation was not present in the manifest zQ175dn mice (males: *r* = 0.08865, *p* = 0.8206; females: *r* = 0.3864, *p* = 0.1548; Pearson’s *r* correlation). Due to the lower weight of manifest zQ175dn mice compared to WT littermates (Fig. 3A), these mice may have an advantage that occludes behavioural deficits caused by the HD mutation. Thus, we normalized for weight in our analysis of time to platform (see methods; non-normalized time to platform data and data separated by sex shown in Additional file 4: Figure S4).

In the acquisition phase, manifest zQ175dn mice had an increased normalized (weight-corrected) time to platform and a higher number of arm entries (Fig. 4B; Additional file 4: Figure S4A, B). Immediately following day 3 of the acquisition phase, all mice performed a probe trial to assess whether they predominantly used an egocentric (striatum-dependent) or allocentric (hippocampal-dependent) strategy [16] to locate the platform. We did not find a significant difference between WT littermates and manifest zQ175dn groups, but all groups showed a greater proportion of mice using an egocentric approach rather than allocentric (Additional file 5: Figure S5A). Of the mice that reached the reversal phase (male WT: 12/15; male manifest zQ175dn: 5/9; female WT: 10/11; female manifest zQ175dn: 14/17), we found a significantly increased normalized time to platform for zQ175dn males and females combined (Fig. 4C) compared to WT mice, and a trend for this in males and females separated (Additional file 4: Figure S4C), but no difference in number of arm entries (Fig. 4C; Additional file 4: Figure S4D).

Premanifest zQ175dn males did not show any difference in time to platform in the acquisition phase, while premanifest females were faster than WT littermates (Additional file 6: Figure S6A). We found a similar pattern for the number of arm entries, with premanifest males showing no genotype effect and premanifest females performing better than WT littermates (Additional file 6: Figure S6B). 60% or more of mice in all groups used an egocentric strategy in the probe trial, as opposed to an allocentric strategy (Additional file 5: Figure S5B). For the mice that reached the reversal phase (male WT: 8/10; male premanifest zQ175dn: 11/12; female WT: 5/9; female premanifest zQ175dn: 8/9), male premanifest zQ175dn mice had a slower time to platform compared to WT littermates but females did not (Fig. 4D), while number of arm entries did not show a genotype difference for either sex (Additional file 6: Figure S6C).

### Time to platform on Water T-Maze is impacted by various behavioural features

We were interested in the finding that male manifest zQ175dn exhibited significantly increased normalized time to platform in the acquisition phase (and a trend for this in the reversal phase), yet these mice did not show any difference in number of arm entries (Additional file 4: Figure S4B). After testing, all mice underwent a swimming speed assessment. We did not find any difference in the swimming speed results between male and female manifest zQ175dn and their WT littermates (male manifest zQ175dn, 19.5 ± 1.25 cm/s, WT littermates, 19.6 ± 1.25 cm/s, *p* = 0.9469; female manifest zQ175dn, 18.5 ± 0.97 cm/s, WT littermates, 18.3 ± 1.69 cm/s, *p* = 0.9081; unpaired t-tests). Therefore, the difference in time to platform did not seem to be due to increased number of arm entries or slower swimming speed. We hypothesized that variability in the time spent at the “decision point”, the area between the arms where mice must turn right or left, could be contributing to differences in time to platform.

We used the software PyMouseTracks [15] to track mouse position in the maze and determine the amount of time spent at the decision point (Fig. 4A). Using this approach, we found a difference in male manifest zQ175dn mice, which spent a larger portion of the trials at the decision point on day 3 of the acquisition phase (portion per trial (%) at decision point: male manifest zQ175dn: 19.4 ± 2.5, WT littermates: 26.1 ± 4.17, interaction effect, *p* = 0.0259; 2-way ANOVA). For the reversal phase, we did not find any significant differences in time at the decision point (across 3-day reversal phase: interaction effect, *p* = 0.6891; 2-way ANOVA). However, if spending more time at the decision point is a contributing factor to longer time to reach the platform, we should find a positive correlation between these two measures. Indeed, we found that normalized time to reach the platform in the acquisition phase was positively correlated with the percentage of time at the decision point in both genotypes (male manifest zQ175dn, *r* = 0.2849, *p* = 0.0104*; WT littermates, *r* = 0.3672, *p* < 0.0001****) and this correlation was stronger when examining day 3 alone (Fig. 4E).

Another factor that could impact time to platform is the number of times an animal stops and restarts in the maze. We analyzed this feature for male manifest zQ175dn mice with PyMouseTracks by counting the number of accelerations per trial in the acquisition phase. We did not find any significant genotype differences in the number of accelerations per trial (male manifest zQ175dn: 6.9 ± 0.50, WT littermates: 7.3 ± 1.5; interaction effect, *p* = 0.2019; 2-way ANOVA). However, we did find a correlation between normalized time to platform and number of accelerations in male WT mice (*r* = 0.3372; *p* < 0.0001****) and the correlation between these two measures was significantly stronger in male manifest zQ175dn mice compared to WT (*r* = 0.5632; *p* < 0.0001****; Fisher’s z test, *p* = 0.022*). On day 3 alone, both genotypes had a strong correlation between number of accelerations and normalized time to platform (Fig. 4F). Altogether, these data show that percentage of time at the decision point and number of accelerations are two behavioural features contributing to time to reach the platform in this task.

### Manifest zQ175dn mice have a significant learning deficit on a lever-pulling task

**Fig. 5:**
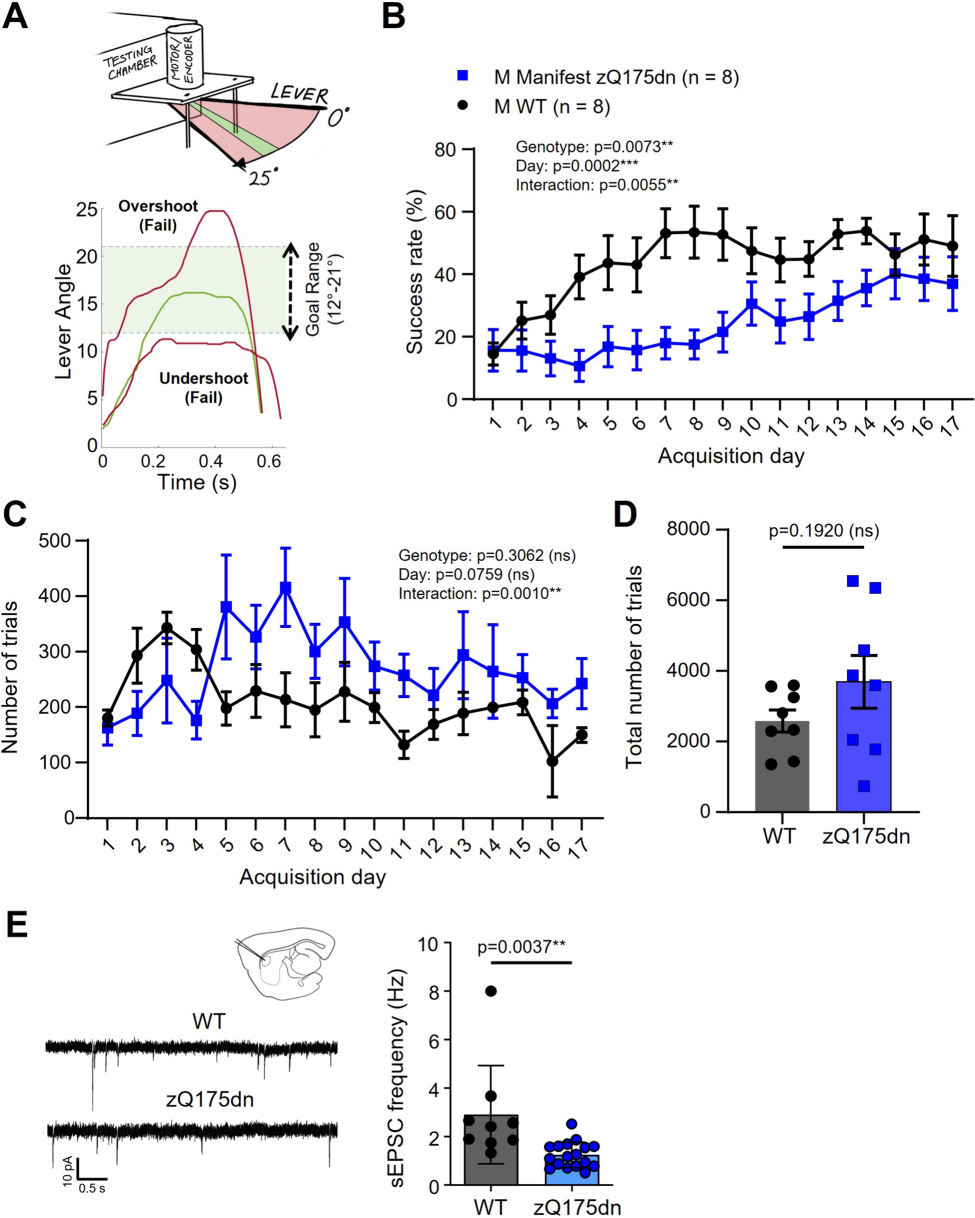
Lever pulling assay and slice electrophysiology in male manifest (10 – 12 month old) zQ175dn mice and wild-type (WT) littermates. **A** Schematic of the testing system of the PiPaw home-cage-based automated testing system used for the lever pulling assay. During the final testing phase, mice must pull the lever into the goal range of 12**°**—21**°** to earn a reward. **B **Success rate per training day in zQ175dn mice compared to WT littermates (mixed effects analysis). **C **Average number of trials performed per day of training (mixed effects analysis). **D T**otal number of trials performed over the entire training period (*p* = 0.1920; unpaired t-test). **E** Spontaneous event frequency for striatal slices harvested from male manifest zQ175dn mice (*n* = 3 animals) and WT littermates (10 – 12 months of age; *n* = 3) previously engaged in the PiPaw task for 3–4 weeks (two-way analysis of variance [ANOVA] with multiple comparisons). Asterisks (*) denote significance level. Individual values for groups with *n* < 6 are provided in Additional file 7: Individual values. ns = not significant. M = Male

Considering the diverse behavioural patterns discerned across our tests, we assessed animals' motor learning and performance on a task requiring more fine motor control. We employed a home-cage strategy—a non-invasive, hands-off approach that reduces experimenter bias. The Pipaw task, a motor control and motor learning assay, was previously employed to evaluate fine motor skills in the Q175FDN strain at 10 months of age (Woodard et al., 2021). Here, we used the same task to assess the zQ175dn mouse model. Group-housed mice had 24/7 access to the task chamber. A trial was deemed successful when a mouse pulled a lever within a designated goal range (Fig. 5A; see methods section). On average, mice attempted 200 to 400 trials daily.

Our analysis, focused on the male manifest zQ175dn group, showed a significant learning deficit compared to WT littermates, as illustrated by a slower rise in success rate over days (Fig. 5B), even though they consistently performed an equal or higher number of daily trials (Fig. 5C). This delayed learning trajectory in the zQ175dn mice eventually reached a performance threshold comparable to that of WT littermates. The average daily trials for both WT and zQ175dn remained consistent over time, suggesting stable motivation to engage with the task (Fig. 5C). Notably, despite the zQ175dn mice engaging in more trials over time, when evaluating the entire learning course, no significant difference was found in the total number of trials between groups (Fig. 5D).

### Reduced spontaneous excitatory activity in acute brain slices from male manifest zQ175dn mice

Building on our behavioural observations, we sought to examine potential neural correlates in striatal activity. Specifically, we aimed to ascertain whether our findings would align with previously reported changes in the spontaneous excitatory postsynaptic currents (sEPSCs) mediated by cortical glutamate release, recorded from spiny projection neurons (SPNs) in acute corticostriatal brain slices from naive HD mice of a similar age [10, 17–19]. This exploration was important for ensuring consistency in our understanding of the disease's progression at the cellular level.

To probe the spontaneous activity of SPNs in the dorsolateral striatum, whole-cell voltage-clamping was employed on sagittal brain slices from male manifest zQ175dn and WT mice that had engaged in the Pipaw lever-pulling task. SPNs were held at -70 mV and frequency and amplitude of sEPSCs were recorded. As expected, zQ175dn mice exhibited lower sEPSC frequency than WT littermates (Fig. 5E). No difference was observed in the amplitude of sEPSCs (WT: 14.81 ± 0.48 pA, *n* = 9(3); zQ175dn: 13.86 ± 0.43 pA, *n* = 17(3)). Our findings corroborate previous observations [10, 19] indicative of consistent alterations in striatal inputs in the progression of HD.

## Discussion

Mouse models of HD have contributed significantly to elucidating mechanisms of mutant Htt toxicity and development of therapeutic approaches to mitigate pathophysiology [3, 4]. Of these models, Q175 heterozygous knock-in mice have been widely used because of their genetic construct validity; however, a better understanding of their stage-dependent behavioural deficits is key to translating molecular-cellular mechanisms towards therapeutic interventions. We identified a variety of behavioural deficits in the Q175 model influenced by age, sex, and mouse background strain. We also determined that differences in the animals’ weight between genotypes can have a considerable effect on behavioural results and describe a way to correct for this. Finally, we used state-of-the-art analysis methods to dissect behavioural phenotypes beyond traditional measures, revealing interesting differences in Q175 mice compared to WT littermates.

### Open Field: Manifest Q175 mice show habituation deficits and changes to behavioural patterns that differ by background strain

Our experiments revealed changes in open field behaviour in Q175 mice as early as 9 months of age. While other studies have shown hyperkinesia at early disease stages and hypokinesia at late stages on similar tasks in HD models [6, 9, 20], findings in the Q175 model have not been as consistent. Deng et al. (2020) found hypokinesia at 6 months of age but not at earlier or later stages in heterozygous zQ175dn mice [20], while other studies have reported decreased distance traveled relative to WT littermates as early as 4 months [6, 9]; however, the latter experiments were performed during the dark phase of the light–dark cycle. During the light phase, Menalled et al. (2012) found no difference in total distance traveled in female heterozygous zQ175dn mice, and only a small decrease in locomotion restricted to 20 weeks of age in males [6]. This is an important consideration, as evidence suggests that zQ175dn mice are hyperactive during the light phase [21], which may have occluded genotype differences in our study.

Consistent with light-phase hyperactivity, male manifest zQ175dn mice, but not females, engaged in rearing behaviour more than WT littermates. In contrast, male and female R6/2 and BACHD mouse models of HD have both been shown to engage in less rearing at manifest ages [22]. However, there are reports of genotype differences in rearing that depend on the phase of the light/dark cycle: YAC128 HD mice showed increased rearing during the light phase, while this was reduced in the dark phase, at 36 and 52 weeks of age [22]; and zQ175dn mice show reduced rearing during the dark but not light phase at 36 weeks of age [6]. Thus, the increase in rearing we observe in this study may be a late-stage phenotype (9—11 months) specific to males during the light phase. Increased rearing may be a perseverative behaviour, and other types of perseverative behaviour have been found in HD models and human patients [5, 23]. Related to this, we found impaired habituation to the open field for male and female manifest zQ175dn mice, similar to previous findings in YAC128 mice [5, 13].

Intriguingly, Q175FDN mice showed reduced locomotor activity at the start of the trial, which could reflect increased anxiety when placed in this novel environment. Other measures of anxiety-like behaviour have been observed in HD models during tasks such as open field, elevated plus maze, fear conditioning, and the light/dark choice test [6, 8, 10, 24, 25]. Notably, a recent study found no difference in total distance traveled or anxiety-like behaviour in the open field in Q175FDN mice [26]. However, this study was performed during the dark phase, thus it is possible that anxiety-like behaviour is more prominent in the light phase.

### Rotarod: performance in manifest zQ175dn mice is impacted by experimental protocol and sex

In male manifest zQ175dn mice, we compared two protocols for the accelerating rotarod task: 1) Rotations Allowed (up to 3 times consecutively) and 2) Standard (ending with a fall or single rotation). With the Rotations Allowed protocol neither zQ175dn nor WT littermates improved on the task, whereas mice of both genotypes improved with training on the Standard protocol. Despite the discrepancy in findings, no prior work to our knowledge has compared these two protocols; moreover, with our set-up, we find that mice frequently rotate instead of falling [13]. Surprisingly, whether rotations are considered equivalent to a fall is not reported in most publications and may contribute to variability in results between studies.

In analysis of paw position over time, we found that WT littermates showed reduced paw slips over days of training, indicating a refinement of motor behaviour, while male (but not female) manifest zQ175dn mice did not. Previously, we observed increased paw slip frequency in male YAC128 mice at early stages even before they developed deficits in latency to fall [13]. Similarly, in this current study we have shown that analyzing paw position over time can reveal behavioural deficits on the rotarod, even when mice seem to be unimpaired or only show subtle deficits by traditional measures.

### Water T-maze: Differential deficits in zQ175dn mice depending on age and sex

During both the acquisition and reversal phases, manifest zQ175dn mice showed increased normalized time to platform. In the YAC128 model, Van Raamsdonk et al. (2005) also reported increased time to platform on day 3 of acquisition on a similar water T-maze task at 8.5 months [5]; those mice also had increased arm entries on day 3 [5], consistent with our observations in manifest zQ175dn mice.

While premanifest zQ175dn mice were able to learn the task similar to or better than their WT littermates, males, but not females, showed an increased time to platform in the reversal phase. YAC128 mice exhibit a reversal learning deficit at 2 months of age, while performing equally well to WT in the acquisition phase [5]. In addition, both the BACHD mouse and BACHD rat models of HD have shown impaired reversal learning in an appetitive version of the T-maze [25, 27]. Deficits in reversal learning may reflect impaired cognitive flexibility, the ability to suppress an old strategy and implement a new one [28]. This is a form of perseverative behaviour, a common symptom in HD patients [29–31].

It is striking that manifest zQ175dn mice only showed a significant impairment for arm entries and had more robust differences in time to platform during the acquisition phase, but not in the reversal phase. One explanation could be selection of the best performers during the acquisition phase, where almost half of the male manifest zQ175dn mice did not meet learning criteria and were excluded from the reversal phase. Our protocol also included fewer trials during task acquisition than other versions of the task [e.g., 5]; implementing a longer acquisition phase may allow more mice to progress to the reversal phase.

To examine the T-maze results more closely, we investigated four possible explanations for deficits in time to platform. Since male manifest zQ175dn mice did not show increased number of arm entries or decreased swimming speed, we explored time at decision point and stopping/starting (accelerations) using PyMouseTracks [15]. For both WT and zQ175dn mice, our analysis provided evidence that these behavioural features contribute to the amount of time mice take to reach the platform, particularly on the final day of the acquisition phase.

### Manifest zQ175dn show impaired learning on the lever-pulling task and reduced spontaneous activity of striatal spiny projection neurons

While it is recognized that tests assessing skilled forelimb use in rodent models possess considerable face and construct validity for evaluating striatal pathology [32, 33], their application in mouse models of HD has so far been scarce. Our previous investigations have used a lever-pulling task to assess forelimb deficits in YAC128 and Q175FDN mouse models [17, 34], as well as a water-reaching task in zQ175dn mice [35]. In the current study, we observed substantial forelimb motor impairments in the home-cage lever-pulling task in male manifest zQ175dn mice. Notably, these mice failed to improve their reward rate on par with WT animals over several weeks of testing.

Although previous literature has suggested that HD mouse models exhibit motivational deficits in operant progressive ratio tasks [36, 37], we found that zQ175dn mice completed an equal or greater number of trials compared to WT mice. This suggests that the motor learning deficits identified here, reminiscent of those observed in the Q175FDN model [17] are unlikely to stem from motivation-related issues. We speculate that the main contributors may be movement planning and/or precise execution, or the effective use of rewards as learning cues.

Slices from mice that performed the lever-pulling assay underwent whole-cell patch clamp recordings from striatal SPNs which revealed a significantly lower spontaneous excitatory event frequency in male manifest zQ175dn mice. These findings are aligned with prior reports of a generally diminished frequency of sEPSCs in the SPNs of zQ175dn mice [19]. Although most electrophysiological studies are conducted in naïve mice [10, 18, 38], the effect of behavioural testing on striatal synaptic activity warrants further research.

### Impact of weight on behaviour in Q175 mice

The impact of animal weight on performance of behavioural assays was a recurring theme in this study. Heterozygous Q175 mice have been widely reported to have lower body weight than WT littermates, and this difference becomes more extreme with age [6, 20, 39]. When we corrected for body weight, we uncovered differences in rotarod and T-maze performance in manifest zQ175dn mice, suggesting that reduced body weight is a confounding factor that masks deficits in zQ175dn mice [20]. In fact, other HD mouse models such as YAC128 and BACHD develop a weight gain phenotype, making them heavier than WT littermates as they age [25, 40], which should also be considered when interpreting results in these models. Normalizing behavioural results by weight or focusing on tasks in which weight does not have a significant effect on performance can address this problem. For example, we observed a robust learning impairment on our home-cage lever-pulling assay in male manifest zQ175dn mice, a task in which lower weight does not provide a perceivable advantage or disadvantage (see [17]).

### Effects of background strain and sex on behaviour in Q175 mice

It is well-known that zQ175dn mice have a slowly progressive phenotype. One solution to this problem is to use enhanced versions of this model with more robust phenotypes, such as Q175FDN mice [10], which we found to have striking differences in open field behaviour compared to WT littermates. Nevertheless, we focused on zQ175dn mice for the present study due to their widespread use.

We uncovered several sex differences in zQ175dn mice, summarized in Table 2. Interestingly, male mice displayed more perseverative behaviours, shown by increased rearing at manifest ages in the open field and impaired reversal learning in T-maze at premanifest ages. Zhang et al. (2020) report abnormal striatal development in male, but not female zQ175dn mice, which may explain some of the deficits we found in males exclusively [41]. More research is needed to elucidate the neural changes responsible for sex-specific behavioural differences in HD mice.

## Conclusions

In addition to exploring behaviour in Q175 knock-in mice, this work has provided key insights for research in general. Firstly, animal weight should be accounted for when analyzing or interpreting results. In addition, the protocol used can greatly impact learning and the ability to detect differences between groups. The phase of the light/dark cycle is also important, as we were not able to replicate some behavioural results found during the dark cycle in previous studies, when conducting our experiments in the light cycle. Our results emphasize the power of using machine learning tools to analyze behaviour, which we used to investigate behaviour in the open field, accelerating rotarod, and water T-maze in deeper ways than traditional methods allow.

Researchers in this field are not only interested in how behaviour is affected in HD, but the underlying neural mechanisms as well. By combining the advanced behaviour analysis tools used in this study with in vivo imaging, we can gain a deeper understanding of HD neuropathology and provide a platform to test therapeutic interventions on both behavioural and neurological phenotypes.

## Methods

### Experimental animals

Experimental animals were group-housed on a 12:12 h light–dark cycle with access to food and water ad libitum, and provided with enrichment including nesting material, a plastic tube, a wooden chew, and a cardboard hut. For the open field, accelerating rotarod, and water T-maze tasks, 2–3 (“premanifest”) and 9–11 month old (“manifest”) male and female Q175 knock-in mice on the C57BL/6 J background strain with excision of the neomycin resistance cassette (zQ175dn; CAG repeat length range: 180–200; obtained from Jackson laboratories: https://www.jax.org/strain/029928) [6] were compared to age-matched C57BL/6 J wild-type (WT) littermates. The sample sizes for each group were: male manifest zQ175dn, *n* = 12, and their WT littermates, *n* = 15; female manifest zQ175dn, *n* = 17, and their WT littermates, *n* = 11; male premanifest zQ175dn, *n* = 12, and their WT littermates, *n* = 10; female premanifest zQ175dn, *n* = 9, and their WT littermates, *n* = 9. A subset of male manifest zQ175dn animals and their WT littermates performed a home-cage lever pulling assay at 10—12 months old (zQ175dn, *n* = 8 of 12; WT, *n* = 8 of 15), and slices were obtained from some of these mice (*n* = 3 for each group) for electrophysiology experiments. Open field experiments were also performed in 10—12 month old (“manifest”) male Q175FDN mice [10], which express a knock-in of the CAG expansion to the mouse locus on the FVB/NJ background strain with the neomycin cassette excised, compared to age-matched FVB/NJ littermates (Q175FDN, *n* = 5; WT, *n* = 6). Table 1 summarizes all behavioural tests and slice experiments performed in this study. All procedures were performed in accordance with the Canadian Council on Animal Care and approved by the UBC Animal Care Committee (Protocols A19-0076; A21-0276; and A23-0083).

**Table 1:**
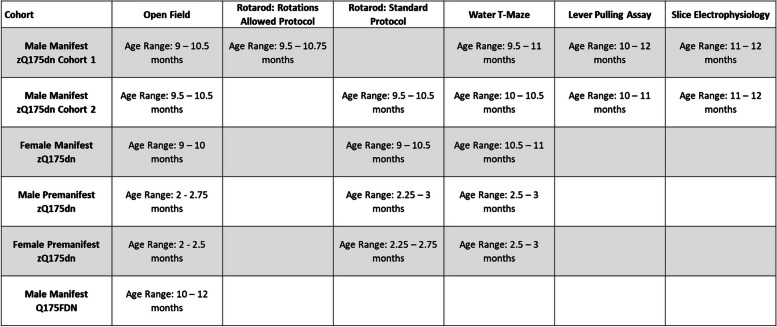
Test age ranges for behavioural work and slice electrophysiology, broken down by cohort

### Behaviour experiments overview

Mice were habituated to the experimenter in the experimental room on two separate days before the first day of testing. On each experiment day, mice were habituated to the experimenter for a few minutes and to the experimental room for one hour prior to behavioural testing. All mice underwent the following experiments in the given order: 1) open field test (1 day), 2) accelerating rotarod (4 days), 3) water T-maze (7 days including 1 break day after day 3), except male manifest Q175FDN groups which only performed the open field test. A subset of male manifest zQ175dn mice and their littermate WT controls (see above) also performed the home cage lever-pulling assay (up to 60 days) after completing the other three tests. All experiments were performed during the light phase of the 12:12 h light–dark cycle. Males and females were tested separately.

### Open field

Mice were placed in a 38 × 38 cm plexiglass arena for 1 h, during which they explored freely. The arena was placed on a clear plexiglass sheet and a raspberry pi camera (PiCamera v1.0 NoIR) was placed below the cage to record the animal’s behaviour. The videos were recorded on a Raspberry Pi 4.

#### Analysis

DeepLabCut was used to track the position of the mouse’s snout, paws, belly, and base of tail (Fig. 1A). Behavioural measures were extracted from this pose information in MATLAB and using the program B-SOiD (https://github.com/YttriLab/B-SOID) [14].

### Accelerating rotarod

For the rotarod task, mice learned to run when placed on a rotating rod (Ugo Basile, Lombardy, Italy) that accelerated from 5–40 rpm over 300 s. The rotarod was stopped by a sensor or manually by the experimenter when the mouse fell. Often, mice rotated around the rotarod instead of falling off. For our first experiments, we allowed mice to continue the trial after rotating, but would stop the trial when a mouse had 3 consecutive rotations, which was treated as equivalent to a fall. In this paper, we refer to this protocol as the “Rotations Allowed protocol”. However, we found that both male WT and zQ175dn mice at 9.5–11 months old did not learn well with this protocol. Therefore, we changed our protocol to consider one rotation equivalent to a fall and stopped the trial the first time a rotation occurred. This protocol (which we refer to as the “Standard protocol”) was used for a second cohort of 9–11-month-old males, as well as all other cohorts in this study. Mice performed 3 trials per day for 4 days with a 1.5—2-h inter-trial interval. A raspberry pi camera (PiCamera v1.0 NoIR) was used to video record the mouse’s hind paws on the rotarod.

#### Analysis

We found that weight had a significant effect on rotarod performance in WT, but not zQ175dn animals, for the manifest age groups only; the 2—3-month-old animals showed no genotype difference in weight, nor any effect of weight on performance within genotype. Therefore, for the 9 – 11-month age groups we performed a weight correction for our analysis of time on rotarod by multiplying time on rotarod by a factor (individual animal weight/average zQ175dn weight). This approach corrected for larger animals having a disadvantage on the rotarod task due to weight that can occlude genotype differences.

We used DeepLabCut [42] to track paw position relative to the rotarod. Data files generated by DeepLabCut were analyzed using in-house-made scripts in MATLAB [13, 43]. A small number of files with disrupted or poor-quality video data were excluded from analysis. Trials shorter than 20 s were excluded from paw slip frequency analysis.

### Water T-Maze

The T-maze apparatus has four arms that form a cross, labeled as North, East, South and West in clockwise order. The North and South arms are ~ 37 cm in length and the East and West arms are ~ 21 cm in length, while all arms are ~ 10 cm in width (Fig. 4A). Animals were placed at the start of the North arm, facing away from the South arm. A sliding door was used to block the South arm, forcing the mice to turn either left or right once they reached the opposite end of the North arm. The T-maze was placed in a small pool and filled with water (24 °C to 30 °C) made opaque by mixing with white acrylic paint to ensure the animals could not see the hidden platform. A raspberry pi camera was mounted above the water maze setup and the video recordings captured the entirety of the T-maze. Mice underwent 3 trials/day for 3 days of the acquisition phase followed by 1 break day and 3 trials/day for 3 days of the reversal phase.

#### Acquisition phase

The position of the platform was randomly assigned for each animal at the start of the acquisition phase and placed in either the East or West arm of the T-maze. The experimenter stirred the water inside the T-maze after each trial to prevent the transfer of olfactory cues between trials. For trials over 30 s, the experimenter guided the mouse to the platform. Trials were separated by an inter-trial interval of at least 45 min. A single probe trial was performed at the end of day 3 to test whether mice used a response learning or a place learning strategy to find the platform during the acquisition phase. During the probe trial, the North arm was blocked instead, and mice were placed in the South arm at the start of the trial with no platform placed in the T-maze. The direction they turned after reaching the end of the South arm was recorded. In one case, the mouse did not choose an arm (2–3-month-old female WT) and was excluded from the probe trial analysis.

#### Reversal phase

Only mice that took just one arm entry to reach the platform (and did not require the experimenter to guide them) for at least 2 of the 3 trials on day 3 of the acquisition phase were included in the reversal phase. For the reversal phase, the position of the platform was reversed (switched from West to East or East to West, depending on which was assigned in the acquisition phase), and the protocol was identical to that used in the training phase. At the end of testing, all mice completed 3 trials of swimming tests to assess differences in swimming speed between genotypes. For each trial, the mouse was placed at the end of the South arm, and the time taken to reach the center of the maze was measured. The average speed of trials 2 and 3 were recorded.

#### Analysis

Time taken to reach the platform and number of arm entries were recorded manually based on the video data. Due to a flash drive error, some videos for trials on days 1 and 2 of the acquisition phase for manifest and premanifest females were lost and excluded from analysis (manifest females: 5 of 10 WT and 2 of 14 zQ175dn excluded; premanifest females: 2 of 10 WT and 2 of 12 zQ175dn excluded). Similar to the rotarod analysis, we used a weight correction for time to reach the platform because 9–11-month-old WT mice showed a positive correlation between animal weight and time to platform. For this calculation, time to platform was divided by a factor calculated for each animal (individual weight/average zQ175dn weight). In addition, we used PyMouseTracks tracking software to analyze the mouse’s time spent in different regions of the T-maze [15].

### Lever pulling assay and RFID tag implantation

We utilized a home-cage-based automated testing system known as PiPaw previously developed by our group [17, 34] (Fig. 5A). To enable automated identification of group-housed mice, a glass Radio Frequency Identification (RFID) capsule (Sparkfun SEN-09416) was implanted subcutaneously in the upper thoracic torso (back of the neck) one week prior to the task as described previously [34]. To build the home-cage, a regular mouse conventional cage was retrofitted with a 3D-printed chamber or “testing module”, giving mice the freedom to access the PiPaw apparatus 24/7. This module incorporated a nose-poke port to access a waterspout and a horizontally movable lever, positioned such that mice would naturally rest their forelimb on it while nose-poking. Water rewards were linked solely to task performance, thereby encouraging mice to engage with the apparatus consistently.

The system automated the task initiation, progress tracking, and dispensing of reward. In the first phase, mice were habituated to nose-poking and lever pulling simultaneously, receiving a water drop after each nose-poke on a fixed 15-min interval. After performing 100 such rewarded nose-pokes, the animals transitioned to the next training stage. In the second phase, a reward was contingent on the mouse pulling the lever beyond 8° of the full 25° lever movement range. After 100 successful lever pulls, the mice entered the final testing phase. Here, they needed to pull the lever with an amplitude falling within a specific range of 12° to 21° from the starting position to earn a reward. Mice were kept in the PiPaw testing environment for a maximum duration of 60 days, or until the completion of 16 ± 2 days in the final testing phase, whichever occurred first. This set period ensured a consistent exposure time across all subjects, providing a fair comparison of their learning and adaptation to the task.

#### Analysis

The PiPaw software [17] automatically compiled data into text files, which were then processed with Python custom scripts (https://github.com/cameron-woodard/PiPaw) [43]. An initial refinement stage filtered out any trials with aberrant timestamps or lever position readings. Following this, data from the main testing phase were organized into 24-h bins for analysis. For each bin, total trial counts and successful trial percentages (success rates) were calculated, providing a daily measure of task performance.

### Electrophysiology

Striatal slices used in electrophysiological experiments were harvested from mice previously engaged in the PiPaw task for 3–4 weeks (WT, *n* = 9 cells [3 animals]; zQ175dn, *n* = 17 [3 animals]). The animals were withdrawn from the PiPaw environment immediately before the slice electrophysiology experiments. Mice were anesthetized using isoflurane, then promptly decapitated. Sagittal slices (270–300 μm) were obtained from left and right hemispheres using a vibrotome (Leica Microsystems, VT1000) in ice-cold artificial cerebrospinal fluid (aCSF). The slices were then transitioned to a chamber with aCSF at 37 °C for a duration of 45 min followed by incubation in aCSF at ambient temperature for at least 30 min before the initiation of whole-cell experiments. Throughout slicing, recovery, and all subsequent procedures, aCSF was constantly oxygenated with carbogen (95% O2/5% CO2). Once in the recording chamber, the slices were consistently bathed in room temperature aCSF supplemented with picrotoxin (50 μM; Tocris Bioscience) to inhibit GABA_A_ receptors and decrease inhibitory responses. All aCSF contained the following (in mM): 125 NaCl, 2.5 KCl, 25 NaHCO_3_, 1.25 NaH_2_PO_4_, and 10 glucose. In addition, aCSF used for cutting slices contained 0.5 mM CaCl_2_ and 2.5 mM MgCl_2_, while all other aCSF contained 2 mM CaCl_2_ and 1 mM MgCl_2_. The pH and osmolarity of the aCSF was adjusted to 7.3–7.4 and 310 (± 3) mOsm/L, respectively.

Recordings were made from striatal spiny projection neurons using the whole-cell patch-clamp technique. The recordings were captured with an Axopatch-700A amplifier, digitized at a sampling rate of 20 kHz (filtered at a 1 kHz with a hardware Bessel filter), and processed with pClamp 11 software. Micropipettes, having a resistance between 3–5 MΩ, were pulled from borosilicate glass capillaries using a micropipette puller (Narishige International). The intracellular solution, based on cesium, was composed of (in mM): 130 cesium methanesulfonate, 5 CsCl, 4 NaCl, 1 MgCl_2_, 5 EGTA, 10 HEPES, 5 QX-314 chloride, 5 MgATP, 0.5 MgGTP, and 10 sodium phosphocreatine. This solution was set to a pH of 7.35, and an osmolarity of 290 (± 3) mOsm/L. Any cells exhibiting a series resistance exceeding 17 MΩ were excluded from the recording process. Spontaneous EPSCs (sEPSCs) were documented by maintaining the cells at a voltage-clamp of -70 mV. For the analysis of electrophysiology data, Clampfit 10.7 (Molecular Devices) was employed.

### Statistics

Plotting of all data and statistical analysis was conducted with Graphpad Prism 10 (San Diego, CA) unless otherwise stated. Male and female data from both genotypes were compared using two-way analysis of variance (ANOVA) and the sexes were combined when no sex differences were found using the statistical test. When sexes were combined, data separated by sex is still presented in supplemental figures and Table 2. Group data were compared using ordinary one-way ANOVA or two-way ANOVA, with Sidak’s test for multiple comparisons. Mixed-effects analysis was used instead of ANOVA when there were missing values. Pairwise comparisons were done using two-tailed unpaired *t* tests. Correlations were calculated using Pearson’s *r* correlation test, and Fisher’s *z* test of correlations was used to compare between correlations [44]. The statistical tests chosen for our analysis were appropriate as our data followed a Normal distribution and had similar variances between groups. The statistical significance was set at *p* < 0.05. All data are presented as mean ± standard error of the mean (SEM). The specific statistical tests used for each experiment, sample size, and *p*-values are reported in the figure legends. In all figures, asterisks indicate statistical significance (* = *p* < 0.05, ** = *p* < 0.01, *** = *p* < 0.001, **** = *p* < 0.0001).

**Table 2:**
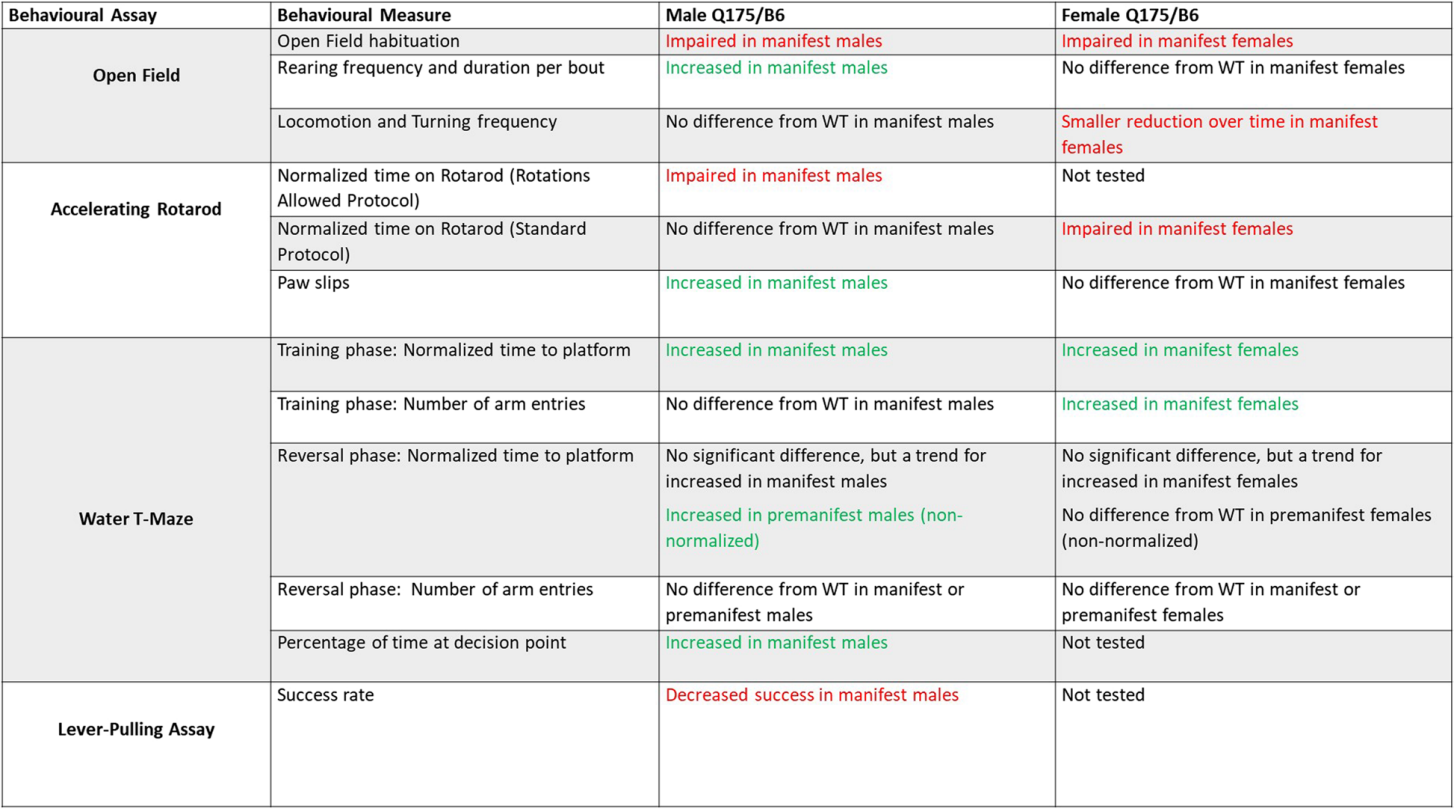
Summary of relevant behavioural results in male and female zQ175dn mice for the open field, accelerating rotarod, water T-maze, and lever-pulling assay

### Supplementary Information

**Additional file 1: Figure S1.** Total distance traveled and percentage change in distance traveled per 5-min intervals during a single 60-min open field trial in zQ175 mice compared to wild-type (WT) littermates. **A**) Male manifest zQ175dn mice. Left: total distance traveled. Right: percentage change in distance. **B**) Female manifest zQ175dn mice. Left: total distance traveled. Right: percentage change in distance. **C**) Left: male premanifest mice – total distance traveled. Right: female premanifest mice – total distance traveled. Two-way analysis of variance was used for all statistical analysis. Asterisks (*) denote significance level. ns = not significant. M = Male. F = Female.

**Additional file 2: Figure S2.** Engagement in behaviours of interest over time during a single open field trial in zQ175 mice compared to wild-type (WT) littermates. **A**) Percentage of time that mice engage in locomotion and turning behaviours over entire trial (top panel) or 5-min intervals across entire trial (bottom 4 panels). **B**) Percentage of time that mice engage in sniffing behaviours. One-way or two-way analysis of variance [ANOVA] with multiple comparisons was used for all statistical analysis unless otherwise noted. Individual values for groups with *n* < 6 are provided in Additional file 7: Individual values. Asterisks (*) denote significance level. ns = not significant. M = Male. F = Female.

**Additional file 3: Figure S3.** Raw and normalized data for accelerating rotarod in male and female manifest zQ175dn mice compared to wild-type (WT) littermates. **A**) Time on rotarod (left) and time to first rotation (right) for male manifest zQ175dn mice performing the Rotations Allowed Protocol (no significant genotype differences; two-way analysis of variance [ANOVA] with multiple comparisons). **B**) Time on rotarod normalized by weight (see methods for details) for male manifest zQ175dn mice performing the Rotations Allowed Protocol (genotype effect, *p* = 0.0322*). **C**) Time on rotarod for male manifest zQ175dn mice performing the Standard Protocol (genotype effect, *p* < 0.0001****, day effect, *p* < 0.0001****). **D**) Time on rotarod for female manifest zQ175dn mice performing the Standard Protocol (no significant genotype differences, day effect, *p* < 0.0001****). Two-way analysis of variance [ANOVA] with multiple comparisons was used for all statistical analysis. M = Male. F = Female.

**Additional file 4: Figure S4.** Normalized (weight-corrected) and raw data for water T-maze in male and female manifest zQ175dn mice compared to wild-type (WT) littermates. **A**) Time to platform normalized by weight during the acquisition phase (males, day 2 difference *p* = 0.0155, day 3 difference, *p* = 0.0097 (multiple comparisons); females, day 3 difference, *p* = 0.0132 (multiple comparisons). **B**) Average number of arm entries during the acquisition phase. **C**) Time to platform normalized by weight during the reversal phase**. D**) Average number of arm entries during the reversal phase. **E**) Time to platform (non-normalized) for male and female manifest zQ175dn in the water T-maze during the acquisition phase. **F**) Time to platform (non-normalized) for male and female manifest zQ175dn in the water T-maze during the reversal phase. Note: due to a flash drive error, 2 female manifest zQ175dn and 5 WT littermates were excluded from analysis for days 1 and 2 of acquisition for water T-maze experiments (therefore for these two days *n* = 15 for female manifest zQ175dn and *n* = 6 for WT littermates). See methods for details. Two-way analysis of variance [ANOVA] with multiple comparisons was used for all statistical analysis. Individual values for groups with n < 6 are provided in Additional file 7: Individual values. ns = not significant. M = male. F = female.

**Additional file 5: Figure S5.** Probe Trial results for the Water T-Maze in manifest and premanifest zQ175dn mice. **A**) Proportion of mice that used an egocentric strategy (as opposed to an allocentric strategy) during the probe trial. No significant differences were found between zQ175dn mice and WT littermates for any groups (M Manifest zQ175dn, *p* = 0.6099; F Manifest zQ175dn, *p* = 0.1650; M Premanifest zQ175dn, *p* = 0.8492; F Premanifest zQ175dn, *p* = 0.3394; unpaired t-tests). M = male. F = female.

**Additional file 6: Figure S6.** Water T-maze performance in premanifest zQ175dn mice and wild-type (WT) littermates. **A**) Average time to platform during the acquisition phase. **B**) Average number of arm entries during the acquisition phase. **C**) Average number of arm entries during the reversal phase. Note: due to a flash drive error, 2 female premanifest zQ175dn and 2 WT littermates were excluded from analysis for days 1 and 2 of acquisition (therefore for these two days *n* = 7 for female premanifest zQ175dn and *n* = 7 for WT littermates). See methods for details. Two-way analysis of variance [ANOVA] with multiple comparisons was used for all statistical analysis unless otherwise noted. Asterisks (*) denote significance level. ns = not significant. Individual values for groups with *n* < 6 are provided in Additional file 7: Individual values. M = Male. F = Female.

**Additional file 7: **Individual values for all datasets with *n* < 6 (and the group they are directly compared to).

## Data Availability

Data generated or analyzed during this study are included in this published article, its supplementary information files, and in publicly available repositories [46–49]. Unpublished MATLAB data analysis code is also available in a publicly available repository [43]. Previously published data analysis software is available at: https://github.com/DeepLabCut/DeepLabCut (DeepLabCut markerless tracking software); https://github.com/YttriLab/B-SOID (B-SOID open field analysis); https://github.com/tf4ong/PMT (PyMouseTracks T-maze analysis); https://github.com/cameron-woodard/PiPaw (PiPaw lever-pulling assay analysis). Individual data values for groups with *n* < 6 can be found in Additional file 7: Individual values.

## Abbreviations

aCSF: Artificial cerebrospinal fluid
ANOVA: Analysis of variance
HD: Huntington’s Disease
Htt: Huntingtin
mHtt: Mutant Htt
RFID: Radio frequency identification
SEM: Standard error of the mean
sEPSC: Spontaneous excitatory postsynaptic current
SPN: Spiny projection neuron
WT: Wild-type
