# Supplementary figures and images for: Deep behavioural phenotyping of the Q175 Huntington disease mouse model: effects of age, sex, and weight
Source: BMC Biol. 2024 May 23;22:121. doi: 10.1186/s12915-024-01919-9 (PMC11119712; doi:10.1186/s12915-024-01919-9)

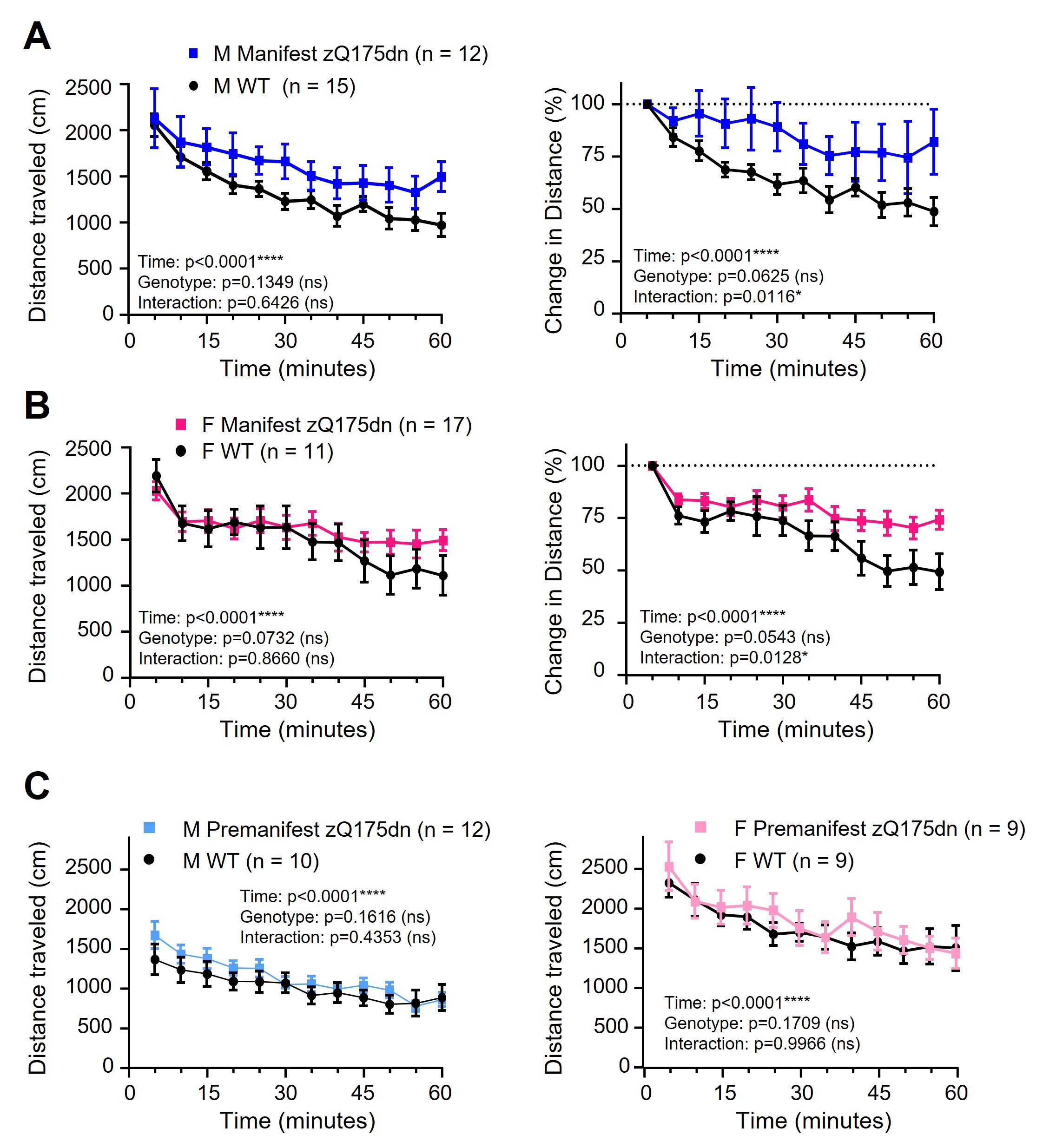

Supplement: Supplementary file 1 — Additional file 1: Figure S1. Total distance traveled and percentage change in distance traveled per 5-min intervals during a single 60-min open field trial in zQ175 mice compared to wild-type (WT) littermates. A) Male manifest zQ175dn mice. Left: total distance traveled. Right: percentage change in distance. B) Female manifest zQ175dn mice. Left: total distance traveled. Right: percentage change in distance. C) Left: male premanifest mice – total distance traveled. Right: female premanifest mice – total distance traveled. Two-way analysis of variance was used for all statistical analysis. Asterisks (*) denote significance level. ns = not significant. M = Male. F = Female. [file 12915_2024_1919_MOESM1_ESM.jpg]

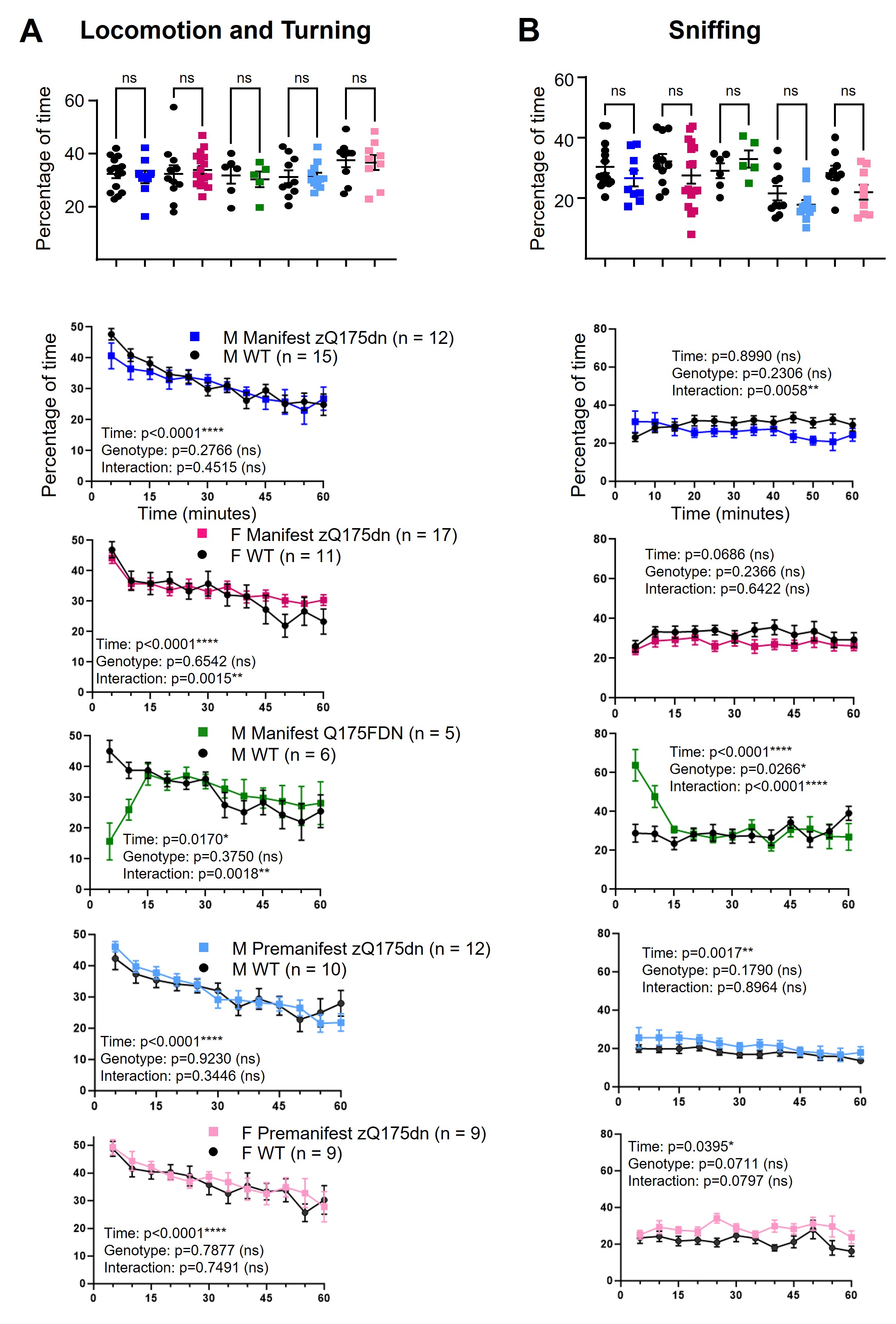

Supplement: Supplementary file 2 — Additional file 2: Figure S2. Engagement in behaviours of interest over time during a single open field trial in zQ175 mice compared to wild-type (WT) littermates. A) Percentage of time that mice engage in locomotion and turning behaviours over entire trial (top panel) or 5-min intervals across entire trial (bottom 4 panels). B) Percentage of time that mice engage in sniffing behaviours. One-way or two-way analysis of variance [ANOVA] with multiple comparisons was used for all statistical analysis unless otherwise noted. Individual values for groups with n < 6 are provided in Additional file 7: Individual values. Asterisks (*) denote significance level. ns = not significant. M = Male. F = Female. [file 12915_2024_1919_MOESM2_ESM.jpg]

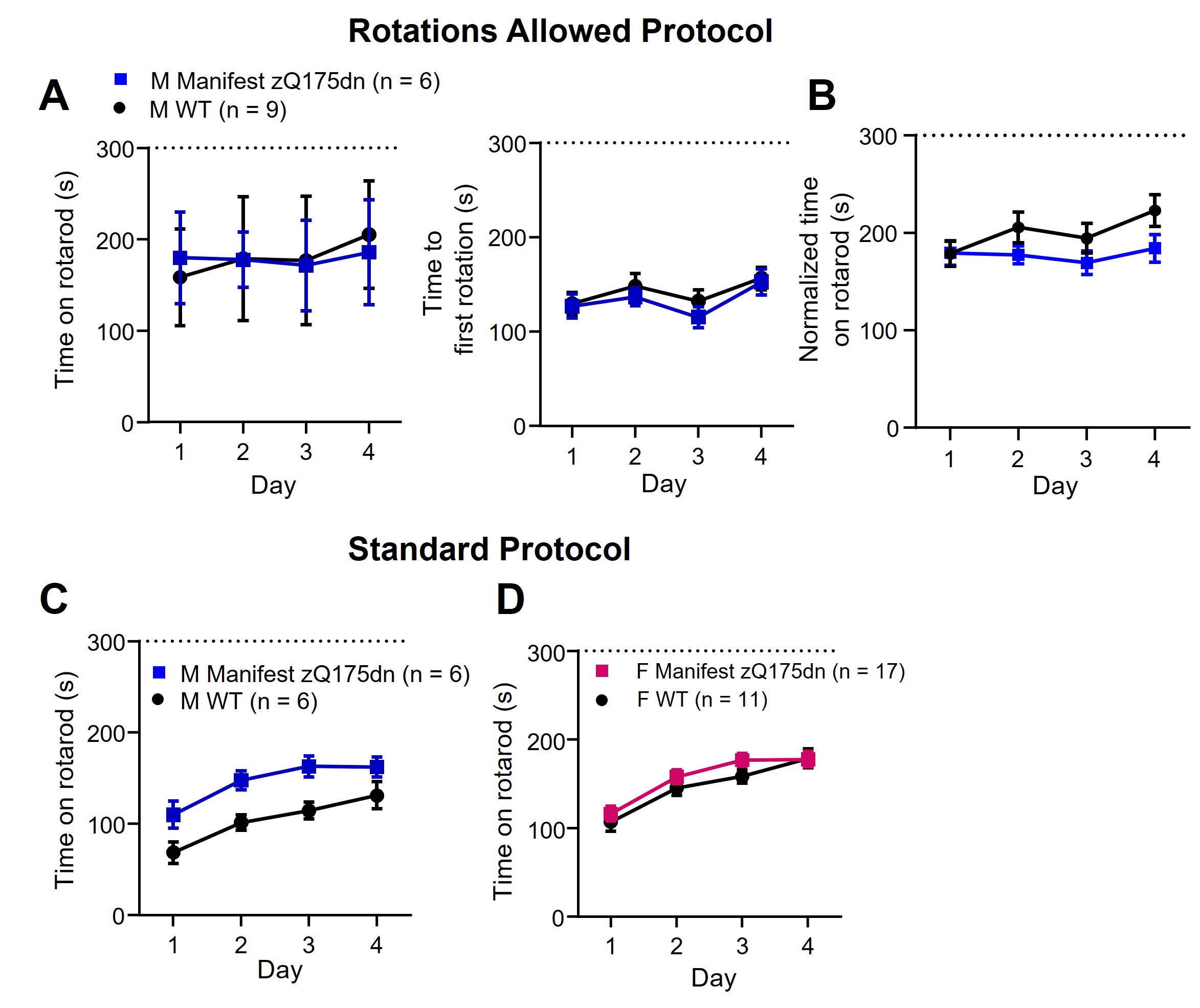

Supplement: Supplementary file 3 — Additional file 3: Figure S3. Raw and normalized data for accelerating rotarod in male and female manifest zQ175dn mice compared to wild-type (WT) littermates. A) Time on rotarod (left) and time to first rotation (right) for male manifest zQ175dn mice performing the Rotations Allowed Protocol (no significant genotype differences; two-way analysis of variance [ANOVA] with multiple comparisons). B) Time on rotarod normalized by weight (see methods for details) for male manifest zQ175dn mice performing the Rotations Allowed Protocol (genotype effect, p = 0.0322*). C) Time on rotarod for male manifest zQ175dn mice performing the Standard Protocol (genotype effect, p < 0.0001****, day effect, p < 0.0001****). D) Time on rotarod for female manifest zQ175dn mice performing the Standard Protocol (no significant genotype differences, day effect, p < 0.0001****). Two-way analysis of variance [ANOVA] with multiple comparisons was used for all statistical analysis. M = Male. F = Female. [file 12915_2024_1919_MOESM3_ESM.jpg]

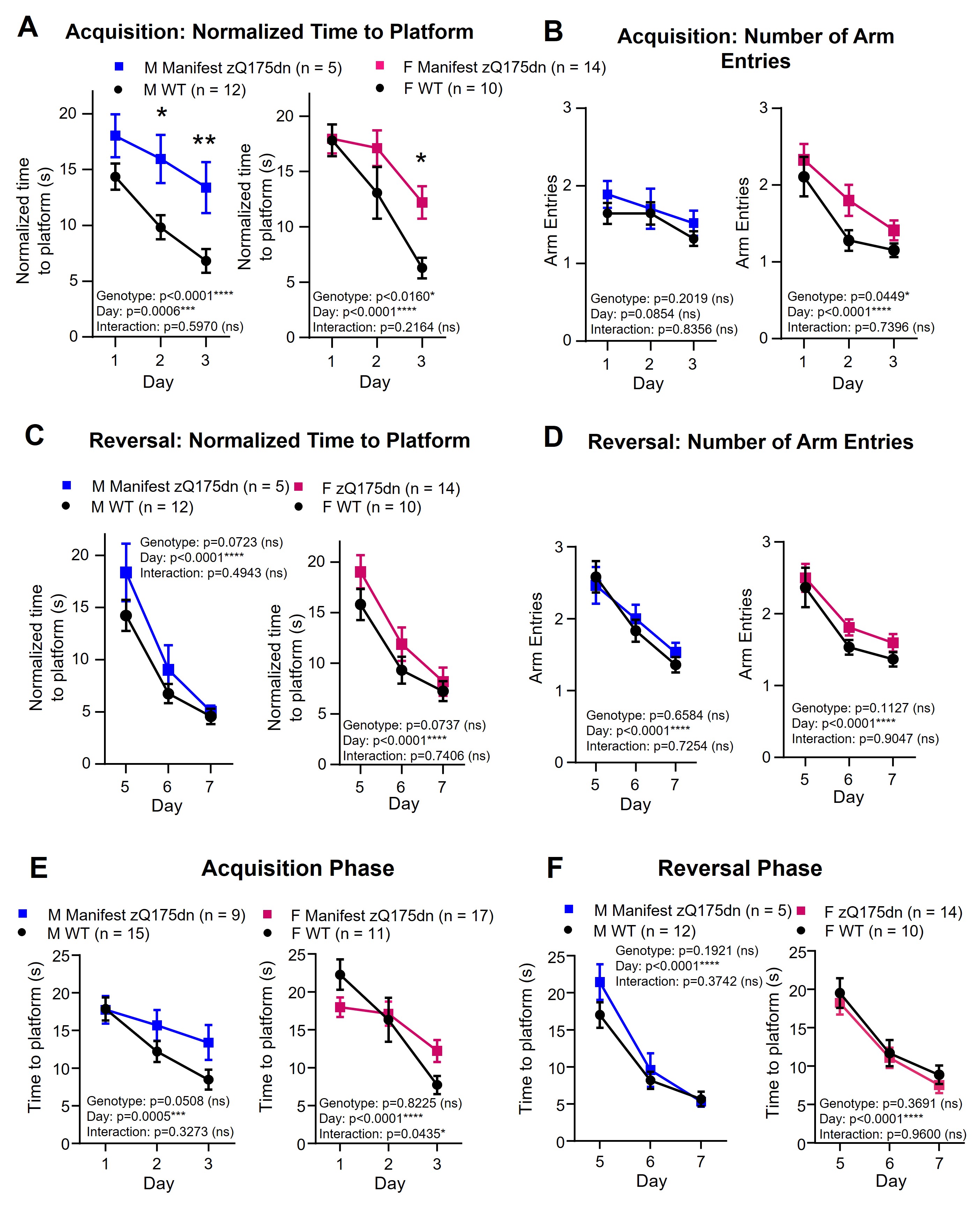

Supplement: Supplementary file 4 — Additional file 4: Figure S4. Normalized (weight-corrected) and raw data for water T-maze in male and female manifest zQ175dn mice compared to wild-type (WT) littermates. A) Time to platform normalized by weight during the acquisition phase (males, day 2 difference p = 0.0155, day 3 difference, p = 0.0097 (multiple comparisons); females, day 3 difference, p = 0.0132 (multiple comparisons). B) Average number of arm entries during the acquisition phase. C) Time to platform normalized by weight during the reversal phase. D) Average number of arm entries during the reversal phase. E) Time to platform (non-normalized) for male and female manifest zQ175dn in the water T-maze during the acquisition phase. F) Time to platform (non-normalized) for male and female manifest zQ175dn in the water T-maze during the reversal phase. Note: due to a flash drive error, 2 female manifest zQ175dn and 5 WT littermates were excluded from analysis for days 1 and 2 of acquisition for water T-maze experiments (therefore for these two days n = 15 for female manifest zQ175dn and n = 6 for WT littermates). See methods for details. Two-way analysis of variance [ANOVA] with multiple comparisons was used for all statistical analysis. Individual values for groups with n < 6 are provided in Additional file 7: Individual values. ns = not significant. M = male. F = female. [file 12915_2024_1919_MOESM4_ESM.jpg]

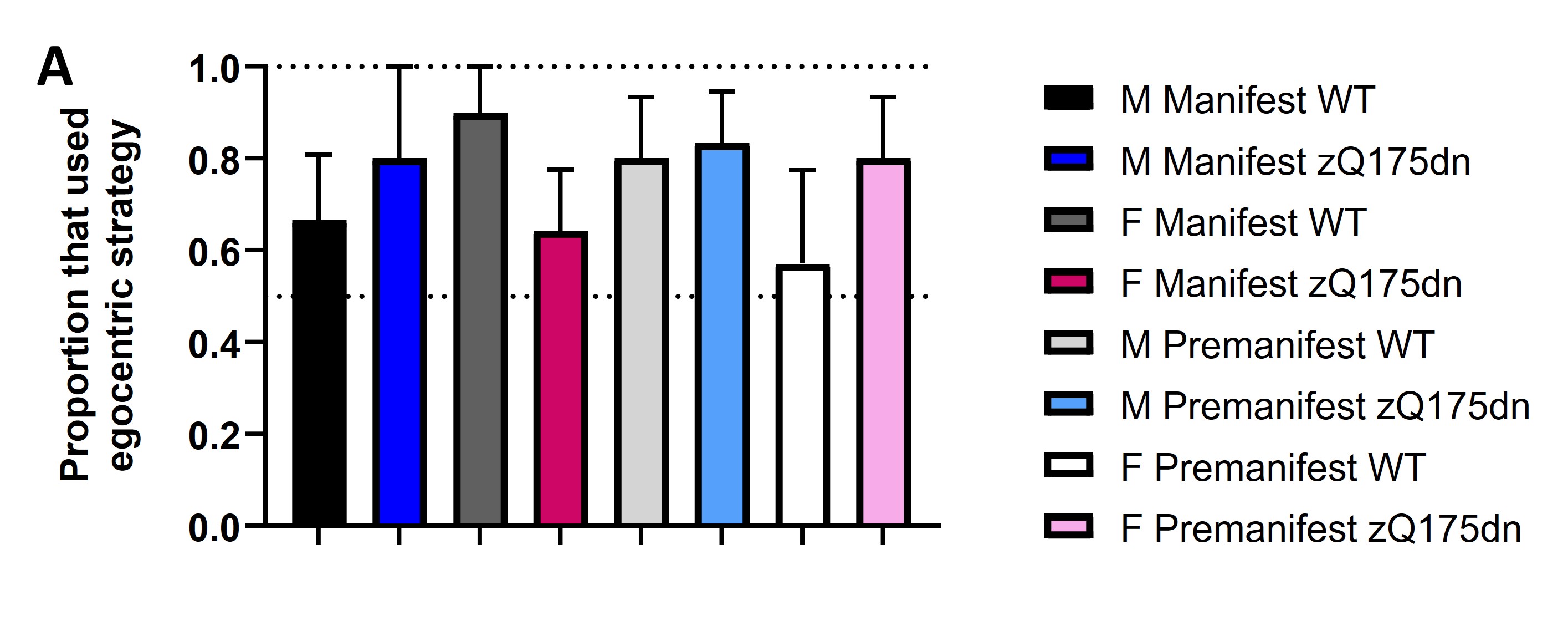

Supplement: Supplementary file 5 — Additional file 5: Figure S5. Probe Trial results for the Water T-Maze in manifest and premanifest zQ175dn mice. A) Proportion of mice that used an egocentric strategy (as opposed to an allocentric strategy) during the probe trial. No significant differences were found between zQ175dn mice and WT littermates for any groups (M Manifest zQ175dn, p = 0.6099; F Manifest zQ175dn, p = 0.1650; M Premanifest zQ175dn, p = 0.8492; F Premanifest zQ175dn, p = 0.3394; unpaired t-tests). M = male. F = female. [file 12915_2024_1919_MOESM5_ESM.jpg]

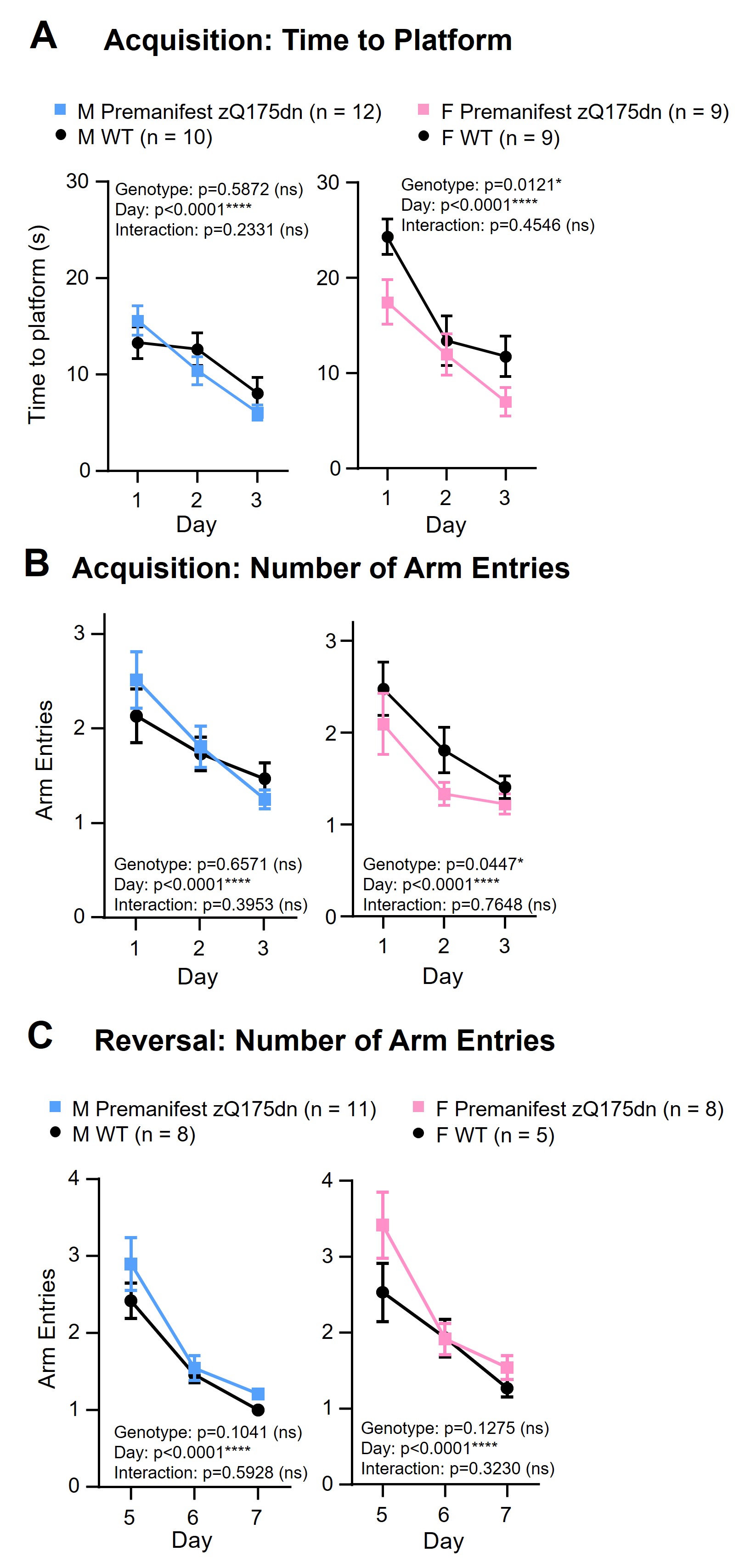

Supplement: Supplementary file 6 — Additional file 6: Figure S6. Water T-maze performance in premanifest zQ175dn mice and wild-type (WT) littermates. A) Average time to platform during the acquisition phase. B) Average number of arm entries during the acquisition phase. C) Average number of arm entries during the reversal phase. Note: due to a flash drive error, 2 female premanifest zQ175dn and 2 WT littermates were excluded from analysis for days 1 and 2 of acquisition (therefore for these two days n = 7 for female premanifest zQ175dn and n = 7 for WT littermates). See methods for details. Two-way analysis of variance [ANOVA] with multiple comparisons was used for all statistical analysis unless otherwise noted. Asterisks (*) denote significance level. ns = not significant. Individual values for groups with n < 6 are provided in Additional file 7: Individual values. M = Male. F = Female. [file 12915_2024_1919_MOESM6_ESM.jpg]
